# Prognostic and predictive impact of *NOTCH1* in early breast cancer

**DOI:** 10.1007/s10549-024-07444-1

**Published:** 2024-08-17

**Authors:** Julia Engel, Vanessa Wieder, Marcus Bauer, Sandy Kaufhold, Kathrin Stückrath, Jochen Wilke, Volker Hanf, Christoph Uleer, Tilmann Lantzsch, Susanne Peschel, Jutta John, Marleen Pöhler, Edith Weigert, Karl-Friedrich Bürrig, Jörg Buchmann, Pablo Santos, Eva Johanna Kantelhardt, Christoph Thomssen, Martina Vetter

**Affiliations:** 1 Department of Gynaecology, Martin Luther University Halle-Wittenberg, Halle (Saale), Germany; 2 Institute of Pathology, Martin Luther University Halle-Wittenberg, Halle (Saale), Germany; 3 Onkologische Gemeinschaftspraxis, Fürth, Germany; 4 Department of Obstetrics and Gynaecology, Klinikum Fürth, Nathanstift Fürth, Germany; 5 Gynäkologisch-Onkologische Praxis, Hildesheim, Germany; 6 Present Address: Frauenärzte Am Bahnhofsplatz, Hildesheim, Germany; 7 Department of Gynaecology, Hospital St. Elisabeth and St. Barbara, Halle (Saale), Germany; 8 Department of Gynaecology, St. Bernward Hospital, Hildesheim, Germany; 9 Department of Gynaecology, Helios Hospital Hildesheim, Hildesheim, Germany; 10 Department of Gynaecology, Asklepios Hospital Goslar, Goslar, Germany; 11 Present Address: Department of Gynaecology and Obstretrics, Hospital Wolfenbüttel, Wolfenbüttel, Germany; 12 Institute of Pathology, Hospital Fürth, Fürth, Germany; 13 Present Address: Gemeinschaftspraxis Amberg, Amberg, Germany; 14 Institute of Pathology, Hildesheim, Germany; 15 Institute of Pathology, Hospital Martha-Maria, Halle (Saale), Germany; 16 Institute of Epidemiology, Biometry and Informatics, Martin Luther University Halle-Wittenberg, Halle (Saale), Germany

**Keywords:** Early breast cancer, Notch, Prognosis, Prediction, Chemotherapy resistance

## Abstract

**Purpose:**

Systemic therapy plays a major part in the cure of patients with early breast cancer (eBC). However, personalized treatment concepts are required to avoid potentially harmful overtreatment. Biomarkers are pivotal for individualized therapy. The Notch signalling pathway is widely considered as a suitable prognostic or predictive marker in eBC. This study aimed primarily at assessing the relationship between *NOTCH1* mRNA expression levels and histopathological features of breast cancer tumors, as well as clinical characteristics of the correspondent eBC patients. As a secondary aim, we investigated the prognostic and predictive value of *NOTCH1* by assessing possible associations between *NOTCH1* mRNA expression and recurrence-free interval (RFI) and overall survival after five years of observation.

**Patients and methods:**

The relative *NOTCH1* mRNA expression was determined in 414 tumour samples, using quantitative PCR in a prospective, multicenter cohort (**P**rognostic Assessment **i**n Routine **A**pplication (PiA), 2009–2011, NCT01592825) of 1,270 female eBC patients.

**Results:**

High *NOTCH1* mRNA expression was detected in one-third of the tumours and was associated with negative hormone receptor status and high uPA/PAI-1 status. In addition, high *NOTCH1* mRNA expression was found to be associated with more RFI related events (adjusted hazard ratio 2.1, 95% CI 1.077–4.118). Patients who received adjuvant chemotherapy and had high *NOTCH1* mRNA expression in the tumour (*n* = 86) were three times more likely to have an RFI event (adjusted hazard ratio 3.1, 95% CI 1.321–7.245, *p* = 0.009).

**Conclusion:**

In this cohort, *NOTCH1* mRNA expression had a prognostic and predictive impact. Tumours with high *NOTCH1* mRNA expression may be less sensitive to cytotoxic treatment and downregulation of the Notch signalling pathway (e.g. by γ-secretase inhibitors) may be valuable for eBC therapy as an individualised treatment option.

**Supplementary Information:**

The online version contains supplementary material available at 10.1007/s10549-024-07444-1.

## Introduction

Breast cancer (BC) is the most common cancer worldwide, with a varied biology and an increasing incidence of 11.6% worldwide (2.26 million new diagnoses per year, https://www.who.int/news-room/fact-sheets/detail/breast-cancer). In addition to clinical and histopathological characteristics that are applicable to the daily routine, the identification of potential biomarkers is pivotal for an improved individualized treatment. The first indication of an oncogenic relevance for Notch signalling in BC was discovered in murine breast tumours. *NOTCH1* and *NOTCH4* have been detected in the mouse mammary tumour virus as target genes for insertion and rearrangement, creating mutations and being involved in epithelial mammary oncogenesis [1, 2]. The Notch signalling pathway has a crucial role in the communication among neighbouring cells regarding tumour initiation, proliferation, dedifferentiation and resistance to apoptosis [3]. Furthermore, this pathway is involved in the regulation of angiogenesis [4], epithelial-to-mesenchymal-transition (EMT) [5], metastasis promotion and drug resistance (reviewed in [6]). The notch transmembrane receptors (*NOTCH1–4*) are activated by one of the five ligands (Serrate-like ligands JAG1-2 and Delta-like ligands 1-,3-,4) [7]. The notch transmembrane receptors (NOTCH1–4) are activated by one of the five ligands (Serrate-like ligands JAG1-2 and Delta-like ligands). Afterwards they are cleaved by metalloproteases of the ADAM family (e.g. ADAM10 or ADAM17) and the ɣ-secretase deliberates the active intracellular cytoplasmic domain fragment (NICD). NCID is transported to the nucleus and linked to the DNA binding protein RBPJκ/CBF-1 (suppressor of hairless/Lag-1) to form a transcription activator complex which induces expression of target genes [8, 9]. Typical target genes of Notch are the transcription factors HES and HEY, c-Myc; oncogene cyclin D1/3 and tumour suppressor p21 [10]. Aberrant activation of the Notch signalling pathway with elevated expression of Notch receptors and ligands leads to the accumulation of NICD and an increased expression of several oncogenic target genes [11].

In one of the first Notch studies on BC patients, nearly 20 years ago [12], authors found a direct relationship between high levels of *NOTCH1* and poor overall survival. The potential prognostic and predictive value of Notch signalling in different cancer entities was outlined in a recent review by Zhou and colleagues [13]. Up to now, publicly available mRNA expression datasets and corresponding survival analyses underline the prognostic value of notch receptors, which is also supported by a small prospective study (*n* = 100) [14]. However, clinical trials or translational studies with reasonable numbers of patients for statistical evaluation are still missing.

Although the clinical care of breast cancer improved overall in the last decades, therapeutic resistance is still an obstacle. A proposed theory for cytotoxic treatment resistance hypothesizes enrichment of breast cancer stem-like cells (BCSCs) in the tumour with different properties, characterised by cell-surface marker CD44+/CD24− or functional markers such as high aldehyde dehydrogenase activity (ALDH+). These cells have high Notch activity, with an elevated mRNA expression of *NOTCH1* or *JAG1*, which contributes to drug resistance and self-renewal of BCSCs [15].

This Notch study was a substudy of the prospective PiA-study (NCT 01592825), that was aimed at confirming the prognostic value of the urokinase-type plasminogen activator (uPA) and its inhibitor type 1 (PAI-1) [16]. High levels of uPA and/or PAI-1 (high: uPA ≥ 3 ng/mg total protein, PAI-1 ≥ 14 ng/mg total protein) in breast tumour tissue predict a poor outcome, as well as a benefit from adjuvant chemotherapy [17]. The basic idea of this Notch study was triggered by the relation of Notch ligand/JAG1 signalling to the transcription factors NFκB and CBF-1, which can control the uPA gene activity in BC. Knockdown of *NOTCH1* mRNA expression led to reduced uPA levels, suggesting that Notch signalling may be involved in the regulation of uPA transcription [18].

Our first aim was to assess the relationship between *NOTCH1* mRNA expression levels and histopathological features of breast cancer tumours, as well as clinical characteristics of the correspondent early breast cancer (eBC) patients. We then investigated the prognostic value of *NOTCH1* by assessing potential associations between *NOTCH1* mRNA levels and recurrence-free interval (RFI) and overall survival (OS).

## Materials and methods

### Study design, patients and tumour characteristics

A multicentre study of 1,270 early, non-metastasised breast cancer patients from five certified breast centres in Germany (January 2009 to December 2011) was designed to confirm prospectively the superiority of uPA/PAI-1 risk assessment as compared to pathological risk assessment. The study was registered as the “PiA-study” (Prognostic assessment in routine Application, NCT 01592825), in accordance with the REMARK recommendations (REporting recommendations for tumour MARKer) [19]. Patients were recruited using the following inclusion criteria: female, invasive, non-metastasised eBC and no second cancer, aged 18 or older, independent of lymph node status, tumour size, grading, receptor status of oestrogen receptor (ER), progesterone receptor (PgR), combined as hormone receptor (HR) status and human epidermal growth factor receptor 2 (HER2). All patients were diagnosed and treated (1,070 with primary surgery, 200 with neoadjuvant chemotherapy, NACT), according to the German AGO Guidelines valid at the respective times [20] (https://www.ago-online.de).

**Fig. 1:**
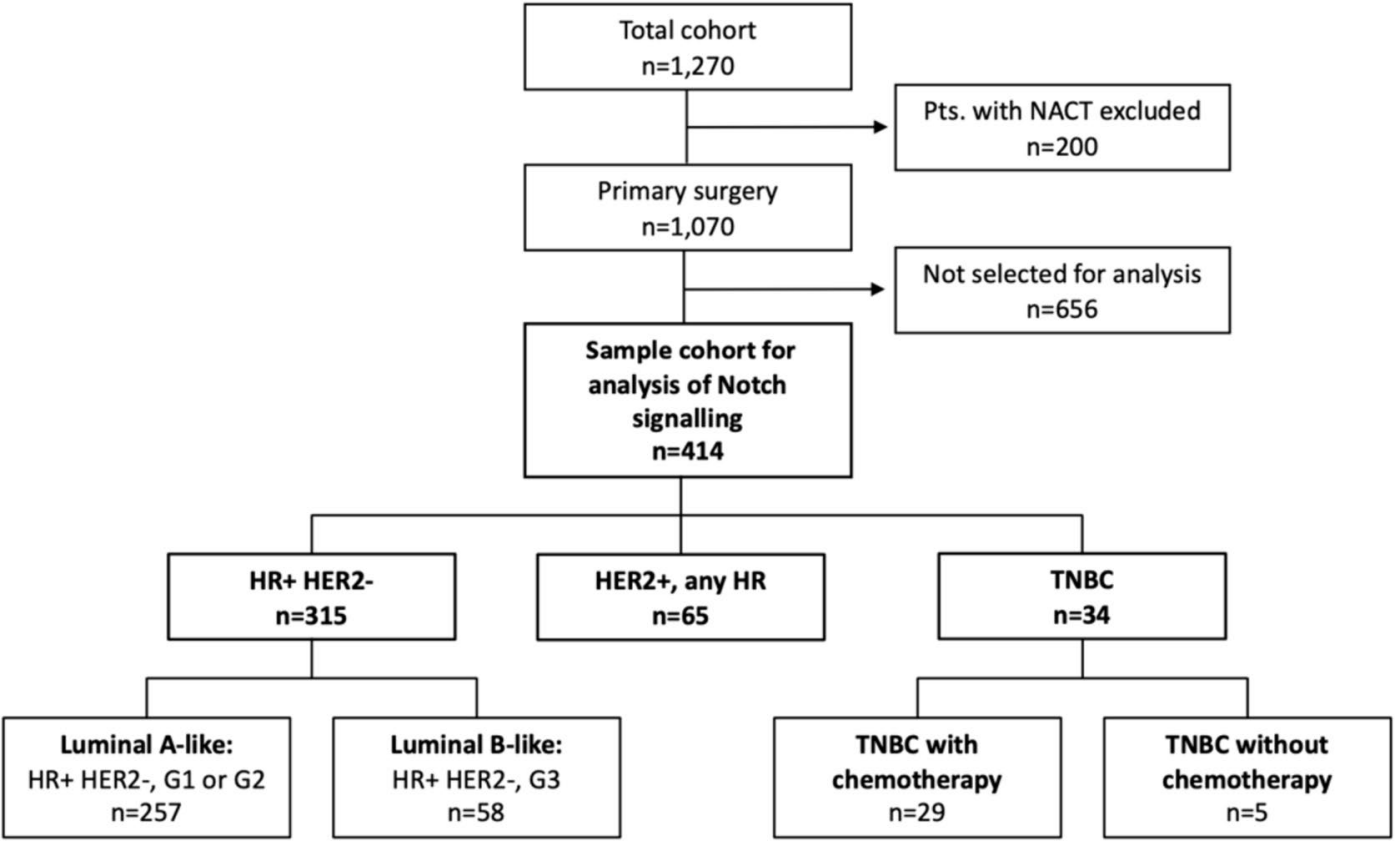
Consort diagram: patients of the PiA-cohort (*n* = 1270) and groups that were used for multivariate *NOTCH1* mRNA expression analyses (*n* = 414). *HR* hormone receptor, *HER2* human epidermal growth factor receptor 2, *NACT* neoadjuvant chemotherapy, *TNBC* triple-negative breast cancer

Here we analysed the *NOTCH1* gene expression in a sample of 414 tumours of all PiA-patients who were enrolled between February 2010 and March 2011, if fresh tissue was available (Fig. 1). We divided the Notch-cohort into three IHC (immunohistochemistry) types: HR-positive/HER2-negative group (*n* = 315), HER2-positive group irrespective of the HR status (*n* = 65) and TNBC (triple-negative breast cancer) group (*n* = 34).

At the time of diagnosis, the median age was 62 years, with 75% of patients older than 50 years. No lymph node involvement was detected in 61% of the patients and 52% of the tumours were larger than 2 cm. For some characteristics, slight differences were observed between the Notch-study cohort and the total PiA-cohort: more high-grade tumours (G3) (26.1% versus 24.3%), and more tumours with a positive HR status (86.2% versus 81.7%) were selected in the Notch-cohort. ER, PgR, HER2 and grading were used for histopathological tumour typing. The main characteristics are summarised in Supplementary Table S1.

### RNA extraction and quantitative PCR analysis

Fresh tumour tissue (50 mg to 200 mg) with a minimum of 40% cancer cells was shock frozen in liquid nitrogen. The tissue was pulverised two times for 45 s at 2000 rpm using Micro-Dismembrator U® (Sartorius AG, Göttingen, Germany). The miRNeasy Mini Kit® (Qiagen GmbH, Hilden, Germany) was applied for RNA isolation. RNA concentration was determined using Infinite M200 Pro Nanoquant (Tecan Trading AG, Männedorf, Switzerland). For cDNA synthesis, 1 µg of total RNA was applied (cDNA Synthesis Kit®, Life Technologies, Carlsbad, CA, USA, Cat number K1642). Quantitative PCR (qPCR) was performed in a Thermo Fisher Scientific StepOne Plus Real-Time PCR system® using TaqMan Gene Expression Assay (Cat number 4453320, RPLP0 ID: Hs99999902_m1, *NOTCH1* ID: Hs00965889_m1*), Master Mix® (Life Technologies, Carlsbad, CA, USA, Cat number 4369016) was used and qPCR protocol was performed according to the manufacturer’s protocol. The thermocycling conditions were 50 °C for 120 s, 95 °C for 20 s, followed by 40 cycles at 95 °C for 1 s and 60 °C for 20 s. Determination of the relative mRNA expression was done by the 2^− ΔΔCT^ method using RPLP0 as a stable constitutively expressed reference gene and a commercially available breast RNA sample for normalisation, as described [21, 22]. In order to ensure the suitability of *RPLP0* as a reference gene, we additionally verified the independence between *RPLP0* expression and disease-related events of patients by assessing the distributions of the qPCR Ct values. Using a Kolmogorov–Smirnov test to compare sample distributions, we found no evidence for an association between disease-related events and *RPLP0* expression (*p* = 0.495, Supplementary Figure S1).

### Statistics

With regard to the first objective, we looked for the distribution of the *NOTCH1* expression levels in relevant clinical and histopathological groups. From logistic regression models, we extracted the odds ratios (OR) and the corresponding 95% confidence intervals (CI). Statistical significance was assessed by using Pearson´s chi-square test (two-sided).

With regard to the second objective, it was necessary to calculate a cut off value with the highest likelihood of a significant separation between high- and low-risk patients. The first endpoint for this objective was recurrence-free interval (RFI: local invasive recurrence, distant recurrence and death from breast cancer), and the second endpoint was overall survival (OS: death from breast cancer, non-breast cancer or unknown causes) according to the STEEP criteria [23]. By the maximum-likelihood method, we calculated the highest chi-square value as the best clinical cut off for RFI, thus dividing the cohort into a patient group with high and one with low risk of recurrence. Restricting the data until the first censored patient, we were able to confirm the cut off by receiver operating characteristic (ROC) analysis including the Youden index (J) method, calculating a cut off value by sensitivity (Se(c)) and specificity (Sp(c)), such that J(c) = {Se(c)-(1-Sp(c))} [24].

The median observation time after diagnosis was 60 months (ranging from 0 to 126). Survival curves for RFI and OS were generated as Kaplan–Meier estimates and data were submitted to the log-rank test. Univariate analyses of RFI and OS were applied to calculate hazard ratios with the corresponding 95% CI. Only clinical and histopathological characteristics with univariate significance were selected for adjustment (Cox regression models) taking into account sufficient events.

The predictive value of *NOTCH1* mRNA expression was determined by exploratory analyses in patient subgroups combining differential *NOTCH1* mRNA expression levels and whether or not they received chemotherapy treatment, visualised in a stacked bar plot.

Statistical analyses were carried out using SPSS 28 (IBM, Armonk, NY, USA).

## Results

### *NOTCH1* mRNA expression and association with clinical and histopathological characteristics

*NOTCH1* mRNA expression was found to be normally distributed across the tumour tissues of the present cohort (Supplementary Figure S2). We found no evidence of a relationship between *NOTCH1* mRNA expression level and the cellularity of the tumour (ratio tumour cells/stromal cells, Supplementary Figure S3).

Considering the median of the relative expression of *NOTCH1* with regard to selected characteristics (Supplementary Figure S4), high mRNA expression was associated with a negative steroid receptor status: 63.5% of the tumours with high *NOTCH1* had a negative ER status (40/63, *p* = 0.020) and 63.2% had a negative HR status (36/57, *p* = 0.032). In addition, associations of *NOTCH1* mRNA expression with the prognostic markers uPA and PAI-1 were observed. Tumours with low uPA and low PAI-1 concentrations (low uPA/PAI-1 status) showed a reduced *NOTCH1* mRNA expression. (90 of 155, 58.1%) and elevated values of uPA and/or PAI-1 were associated with high *NOTCH1* mRNA expression (142/259, 54.8%, *p* = 0.011). Furthermore, we showed that the *NOTCH1* mRNA expression and uPA protein concentration were correlated (Supplementary Figures S5 and S6). No association was detected between *NOTCH1* mRNA expression and age, nodal status, tumour size, grading, PgR and HER2. Considering the clinical outcome with regard to disease-related events (RFI), a cut off value of 2.4 relative mRNA expression was determined to dichotomise the cohort in patients with low (69.3%) and high (30.7%) risk of recurrence (Table 1). Further characteristics of patients and tumours in relation to *NOTCH1* mRNA expression are reported in Table 1 and Supplementary Table S2. Using this cut off value, tumours with high *NOTCH1* mRNA expression were more likely HR negative (OR 2.1, 95% CI 1.20–3.77, *p* = 0.009, Supplementary Table S3) and more likely HER2 positive (OR 3.3, 95% CI 1.89–5.61, *p* < 0.001) than those with low expression. Tumours with high *NOTCH1* mRNA expression were associated with high values of uPA and/or PAI-1 (OR 1.9, 95% CI 1.20–2.98, *p* = 0.006).

**Table 1:** Distribution of low and high *NOTCH1* mRNA expression in selected groups

Characteristics	Notch-cohort	*NOTCH1* low		*NOTCH1* high	
All	*n*	*n*	(%)	*n*	(%)
	414	287	(69.3)	127	(30.70)
Age in yrs					
< 50	104	77	(74.0)	27	(26.0)
≥ 50	310	210	(67.7)	100	(32.2)
Nodal status					
Negative	251	175	(69.7)	76	(30.3)
Positive	163	112	(68.7)	51	(31.3)
Tumour histology					
Ductal (NST)	329	220	(66.9)	109	(33.1)
Lobular	67	52	(77.6)	15	(22.4)
Others	18	15	(83.3)	3	(16.7)
Tumour size					
≤ 2 cm	198	130	(65.7)	68	(34.3)
> 2 cm	216	157	(72.7)	59	(27.3)
Grading					
G1	39	27	(69.2)	12	(30.8)
G2	267	189	(70.8)	78	(29.2)
G3	108	71	(65.7)	37	(34.3)
ER status					
Positive (≥ 1%)	351	**254**	**(72.4)**	**97**	**(27.6)**
Negative (< 1%)	63	**33**	**(52.4)**	**30**	**(47.6)**
PgR status					
Positive (≥ 1%)	297	**214**	**(72.1)**	**83**	**(28.0)**
Negative (< 1%)	117	**73**	**(62.4)**	**44**	**(37.6)**
HR status					
Positive (ER and/or PgR ≥ 1%)	357	**256**	**(71.7)**	**101**	**(28.3)**
Negative (ER and PgR < 1%)	57	**31**	**(54.4)**	**26**	**(45.6)**
HER2 status					
Positive (DAKO 2 if ISH positive, DAKO 3)	65	**30**	**(46.2)**	**35**	**(53.9)**
Negative (DAKO 0, 1 or 2 if ISH negative)	349	**257**	**(73.6)**	**92**	**(26.4)**
Biological tumour types					
Luminal A-like: HR + HER2-, G1 or G2	257	**192**	**(74.7)**	**65**	**(25.3)**
Luminal B-like: HR + HER2-, G3	58	**45**	**(77.6)**	**13**	**(22.4)**
HER2 + , any HR	65	**30**	**(46.2)**	**35**	**(53.9)**
TNBC	34	**20**	**(58.8)**	**14**	**(41.2)**
uPA/PAI-1 status					
Low: uPA and PAI-1 low	155	**120**	**(77.4)**	**35**	**(22.6)**
High: uPA and/or PAI-1 high	259	**167**	**(64.5)**	**92**	**(35.5)**

*ER* oestrogen receptor, *PgR* progesterone receptor, *HR* hormone receptor, *HER2* human epidermal growth factor receptor 2, *ISH* in situ hybridisation, *TNBC* triple-negative breast cancer, *uPA* urokinase-type plasminogen activator cut off ≥ 3 ng/mg total protein, *PAI-1* plasminogen activator inhibitor type 1 cutoff ≥ 14 ng/mg total protein; bold: *p*-value (Pearson *χ*^2^ test) < 0.05

### Survival analysis

**Fig. 2:**
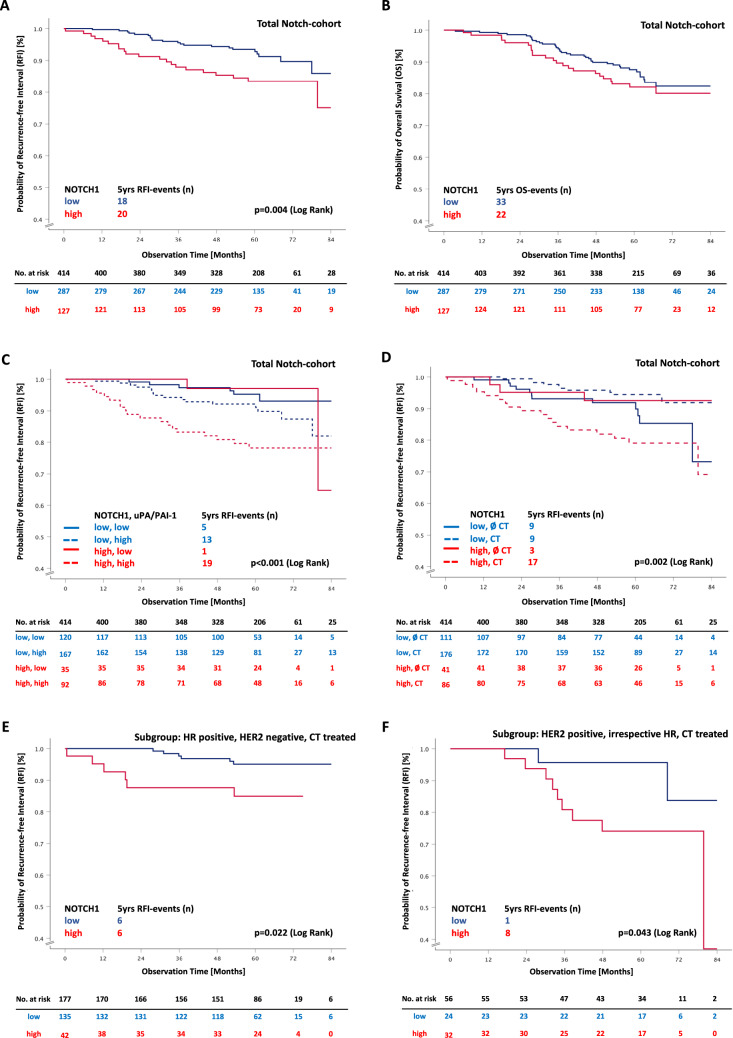
Survival estimates for patients of the Notch-cohort for total Notch-cohort considering *NOTCH1* RFI (**A**) and OS (**B**); total Notch-cohort considering *NOTCH1* and uPA/PAI-1 RFI (**C**); total Notch-cohort considering *NOTCH1* and CT RFI (**D**), HR positive, HER2 negative, CT-treated RFI (**E**); HER2 positive, irrespective HR, CT-treated RFI (**F**); the tables present the effective sample size for each interval (No. at Risk). *uPA* urokinase-type plasminogen activator, *PAI-1* plasminogen activator inhibitor type 1, *uPA/PAI-1 low* uPA and PAI-1 low, *uPA/PAI-1 high* uPA and/or PAI-1 high, *CT* chemotherapy, *HR* hormone receptor, *HER2* human epidermal growth factor receptor 2

Considering the second objective, high *NOTCH1* mRNA expression was associated with more disease-related events and a reduced overall survival probability. Patients with high *NOTCH1* mRNA expression had a 2.5 higher risk of recurrence (95% CI 1.31–4.69), corresponding to a decreased five-year RFI event-free probability of 83.4% (95% CI 76.74–90.06) in comparison to 93.5% (95% CI 90.56–96.44) among patients with low *NOTCH1* mRNA expression (Fig. 2A, Supplementary Table S4A). After five years, 87.6% (95% CI 83.48–91.72) of the patients with low *NOTCH1* mRNA expression were still alive, and 82.1% (95% CI 75.24–88.96) in the group with high *NOTCH1* mRNA expression (Fig. 2B, Supplementary Table S4B, S5). In a multivariate analysis with regard to RFI, including nodal status and biological tumour types, we observed a hazard ratio of 2.1 (95% CI 1.08–4.12,) for patients with *NOTCH1* mRNA overexpressed tumours. Overall, we did not observe any significant association of *NOTCH1* mRNA expression with OS, neither in univariate nor in multivariate analyses (Table 2, Supplementary Table S4B, S5).

**Table 2:** Multivariate analyses of RFI and OS according to *NOTCH1* mRNA expression (*n* = 414) in selected groups

Groups	*n*	Recurrence-free interval, 5 years			Overall survival, 5 years		
		Events	Hazard ratio	95% CI	Events	Hazard ratio	95% CI
ALL^a^							
* NOTCH1* low	287	18	1		33	1	
* NOTCH1* high	127	20	**2.1**	1.08–4.12	22	1.3	0.75–2.30
ALL with regard to CT^b^							
* NOTCH1* low, no CT	111	9	**2.9**	1.10–7.39	27	**7.1**	3.26–15.25
* NOTCH1* low, CT	176	9	1		11	1	
* NOTCH1* high, no CT	41	3	2.3	0.62–8.83	5	**3.6**	1.21–10.86
* NOTCH1* high, CT	86	17	**3.1**	1.32–7.25	18	**2.7**	1.20–6.16
ALL with regard to NOTCH1 and uPA/PAI-1^c^							
* NOTCH1* low, uPA/PAI-1 low	120	5	1		6	1	
* NOTCH1* low, uPA/PAI-1 high	167	13	1.8	0.70–4.63	26	**2.6**	1.12–6.02
* NOTCH1* high, uPA/PAI-1 low	35	1	1.1	0.23–5.58	3	1.6	0.41–6.08
* NOTCH1* high, uPA/PAI-1 high	92	19	**3.7**	1.45–9.33	19	**3.1**	1.31–7.50
HR positive, HER2 negative and CT^d^							
* NOTCH1* low	135	6	1		5	1	
* NOTCH1* high	42	6	**3.6**	1.16–11.17	6	**4.0**	1.22–13.11
HER2 positive, any HR and CT^e^							
* NOTCH1* low	24	1	1		2	1	
* NOTCH1* high	32	8	6.6	0.82–52.61	8	3.2	0.68–14.99

^a^adjusted to *NOTCH1*, nodal status and histopathological groups, ^b^adjusted to *NOTCH1*, nodal status, HR status and HER2 status, ^c^adjusted to *NOTCH1*, uPA/PAI-1, nodal status and grading, ^d^adjusted to *NOTCH1* and nodal status, ^e^adjusted to *NOTCH1* and HR status

*CT* chemotherapy, *HR* hormone receptor, *HER2* human epidermal growth factor receptor 2, *TNBC* triple-negative breast cancer, *uPA* urokinase-type plasminogen activator, *PAI-1* plasminogen activator inhibitor type 1, *uPA/PAI-1 low* uPA and PAI-1 low, *uPA/PAI-1 high* uPA and/or PAI-1 high, *CI* confidence interval; bold: *p*-value (Pearson *χ*^2^ test) < 0.05

Concerning clinically relevant subgroups, we observed that *NOTCH1* mRNA overexpression was associated with a significantly less favourable outcome in patients with HR-positive/HER2-negative tumours getting adjuvant chemotherapy (adjusted hazard ratio 3.6, 95% CI 1.16–11.17, *p* = 0.027). A similar but not significant observation was obtained for patients with HER2-positive tumours (adjusted hazard ratio 3.3, 95% CI 0.70–15.30, Supplementary Table S4A) and in TNBC patients (multivariate analysis was not feasible due to small TNBC sample size).

Following the main prospective aim of the PiA-study, the clinical value of the biomarker uPA/PAI-1, we also assessed the impact of the combination of *NOTCH1* and uPA/PAI-1. The highest risk of recurrence, as well as the poorest overall survival, was detected for the patient group with high *NOTCH1* mRNA expression combined with high uPA/PAI-1 (Fig. 2C and Supplementary Figure S7A). Within five years of follow-up, 21% of these patients experienced an RFI event compared to 4% with low *NOTCH1* mRNA expression and low uPA/PAI-1 (adjusted hazard ratio 3.7, 95% CI 1.45–9.33, *p* = 0.006). Overall survival probability was 78.5% (95% CI 69.88–87.12) for patients with a high *NOTCH1* mRNA expression combined with high uPA/PAI-1 compared to 94.1% (95% CI 89.40–98.80) with low *NOTCH1* mRNA expression and low uPA/PAI-1 status. The adjusted hazard ratio was 3.1 (95% CI 1.31–7.50, *p* = 0.010) (Table 2).

### Predictive value of *NOTCH1*

**Fig. 3:**
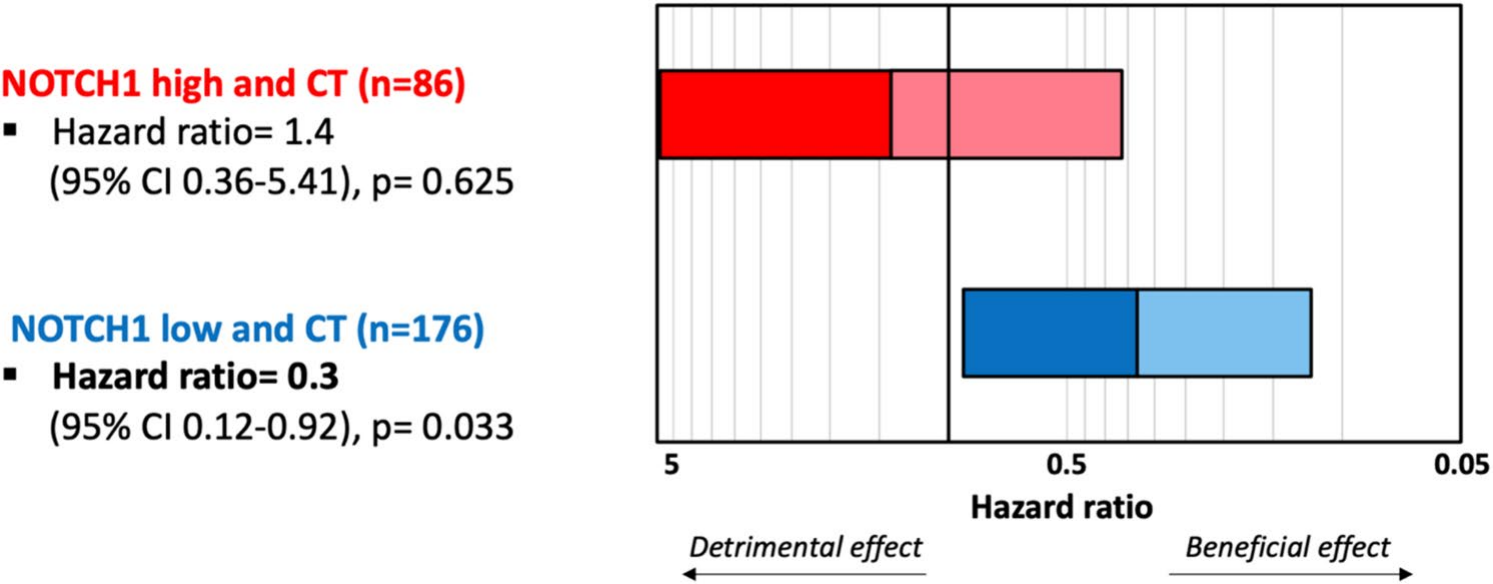
Benefit of chemotherapy by *NOTCH1* low expression visualised as stacked bar graph (blue) Hazard ratios for CT by high and low *NOTCH1* expression (no CT as reference) CT chemotherapy, CI confidence interval

Furthermore, we investigated the predictive impact of *NOTCH1* mRNA expression. In our cohort, we worked out a three-times higher benefit from chemotherapy for patients with low *NOTCH1* mRNA expression compared to patients with high *NOTCH1* mRNA expression (hazard ratio 0.3, 95% CI 0.12–0.92, *p* = 0.033) (Fig. 3).

In addition, we observed that patients with high *NOTCH1* mRNA expression and adjuvant chemotherapy had an unfavourable course of disease (79.1% 5 years RFI event-free probability, 95% CI 70.28–87.92) (Fig. 2D). Patients with high *NOTCH1* mRNA expression and no chemotherapy (92.6%, 95% CI 84.56–99.99), as well as those with low *NOTCH1* mRNA expression with (94.4%, 95% CI 90.87–97.93) or without (89.9%, 95% CI 83.43–96.37) adjuvant chemotherapy, had a more favourable outcome (Fig. 2D, Supplementary Table S4A). In summary, patients with high *NOTCH1* mRNA expression and adjuvant chemotherapy suffered from more recurrences than those without chemotherapy (21.9% vs 7.3%), resulting in an around 3-times increased relative risk for an RFI event, after adjustment to nodal status, hormone receptor status and HER2 status (adjusted hazard ratio 3.1, 95% CI 1.32–7.25).

For patients with HR-positive/HER2-negative tumours, who were treated with adjuvant chemotherapy (*n* = 177), we observed similar results. Patients with high *NOTCH1* mRNA expression experienced more RFI events (6 of 42, 15%) compared to patients with low *NOTCH1* mRNA expression (6 of 135, 5%; adjusted hazard ratio 3.6, 95% CI 1.16–11.17, *p* = 0.027) (Fig. 2E, Supplementary Table S4A). In contrast, for patients with HR-positive/HER2-negative tumours without adjuvant chemotherapy, no impact of *NOTCH1* mRNA expression was observed. Similarly, 25% of patients (8 of 32) with HER2-positive tumours (irrespective of HR status), treated with trastuzumab and chemotherapy (*n* = 56), and high *NOTCH1* mRNA expression experienced an RFI event, and only 4% of patients (1 of 24) with low *NOTCH1* mRNA expression (adjusted hazard ratio = 6.6, 95% CI 0.82–52.61) (Fig. 2F, Supplementary Table S4A). For TNBC patients who received chemotherapy, the same trend was observed. Again, because of the small sample size (*n* = 29), multivariate analyses were not feasible (Supplementary Figure S8A and S8B). Due to the lack of untreated patients, the predictive impact of *NOTCH1* overexpression cannot be formally assessed in HER2-positive and triple-negative breast cancer.

## Discussion

Considering our primary objective, we found that *NOTCH1* mRNA expression in breast tumour tissue follows a normal distribution in an unselected cohort of eBC patients with a median observation time of five years. In line with current knowledge, also in our cohort, the *NOTCH1* mRNA expression is higher in breast tumour tissue than in normal breast tissue and is significantly associated with a more aggressive tumour biology (negative HR status, positive HER2 status and high uPA/PAI-1 status). Studies have shown that the expression of Numb, a negative Notch regulator, is elevated in normal breast tissue and is considerably less expressed in breast cancer tumour tissue, which results in aberrant activation of the Notch signalling pathway [25]. Stylianou and colleagues [11] investigated the activity of Notch signalling in human breast tumour cells and its impact on cellular transformation. They observed an accumulation of NICD, the intracellular domain and active form of Notch, in breast cancer cell lines (e.g. MDA MB 231, MCF7) and tissue samples, whereas there was a loss of Numb expression. .They further generated a normal breast epithelial cell line MCF-10A with elevated levels of NICD that caused the expression of several Notch target genes, increased cell growth, changes in cell shape, and resistance to apoptosis. In addition, in experiments with colorectal cancer (CRC) cell lines it has been described that upregulation of Notch receptors, Notch ligands and Notch target genes is associated with epithelial-mesenchymal transition and formation as well as preservation of cancer stem cell populations and the acquisition of a metastatic phenotype leading to poor survival in patients with CRC [26].

The secondary objectives of our study were the evaluation of the prognostic and the predictive impact of *NOTCH1*. To our knowledge, very few publications had focused on the association of *NOTCH1* with disease-related survival data before. Nearly 20 years ago, Reedijk and colleagues [12] analysed a cohort of patients with early breast cancer (*n* = 184) and detected by univariate analysis a worse ten-year OS probability of 49% in patients with high NOTCH1 protein expression, compared to 64% with low NOTCH1 protein expression (via IHC).

Our data are in line with a recently published meta-analysis of 21 studies including 3,867 patients that confirmed the prognostic impact of *NOTCH1* mRNA expression concerning RFS and OS [27]. This meta-analysis showed a correlation of *NOTCH1* mRNA expression with invasive ductal carcinoma, lymphatic metastasis and histological grading. Furthermore, there was an association of *NOTCH1* with basal type of breast cancer (OR = 2.53, 95% CI 1.18–5.43, *p* = 0.009). In addition, they presented survival curves based on available public mRNA expression datasets (for RFS GSE25066; OS: GSE20685) that showed an association of high *NOTCH1* mRNA expression with worse clinical outcome (RFS, *p* = 0.023 and OS, *p* = 0.042). The limitation of this pooled analysis is that most of these studies were not prospective and less than half of the 21 studies considered *NOTCH1* mRNA expression as the primary endpoint [27]. However, in concordance with the aforementioned pooled analysis, also in our study, patients with high *NOTCH1* mRNA expression had a worse course of disease with shorter RFIs (10% absolute difference for RFI events) and an impaired OS. By using an optimal cutoff for *NOTCH1* mRNA expression, we found a high risk of recurrence for one third of the patients although they have been treated with the best available therapies of the respective time.

In addition, we observed that *NOTCH1* mRNA expression might refine the prognostic value of uPA/PAI-1 by identifying patients with a low risk of relapse more efficiently. With uPA/PAI-1 alone, only 40% of patients were classified as low risk; however, including *NOTCH1* mRNA expression, this proportion could be increased up to 78%, still experiencing less than 10% 5-year disease-related events.

In summary, overall we found a relevant and significant prognostic impact of *NOTCH1* mRNA expression, which was confirmed by multivariate analyses after adjustment to nodal status, grading, ER, PgR, HER2 and uPA/PAI-1. High *NOTCH1* mRNA expression is associated with high risk of recurrence.

With regard to predictive impact, one-third of the patients of our cohort with high tumour *NOTCH1* mRNA expression who were treated with adjuvant chemotherapy experienced relapses within the first five years after diagnosis and had a worse OS probability compared to patients without cytotoxic treatment. This observation may indicate resistance to chemotherapy or even harm from therapy. Regarding the relationship between Notch signalling and breast cancer treatment, we observed a three-times higher benefit from chemotherapy for patients with low *NOTCH1* mRNA expression in the tumour tissue. Other authors like Yao et al. [28] analysed the association of *NOTCH1* mRNA expression with histopathological characteristics, and from that relation, in addition to a potential prognostic impact, they also extrapolated an association to treatment resistance.

With regard to histopathological subtypes, HR-positive/HER2-negative tumours with high *NOTCH1* mRNA expression were associated with chemotherapy resistance. This is in line with published data [29] denoting the potential importance of notch pathway inhibition. We assume that this is also true for HER2-positive eBC and TNBC. Our data fit well with a previously published analysis of TCGA data demonstrating that chemosensitivity is also reduced in TNBCs with high *NOTCH1* mRNA expression as shown in vitro and by survival estimates [30].

High uPA/PAI-1 is associated with enhanced benefits from chemotherapy [31]. In our exploratory analysis, we demonstrated for tumours with a high uPA/PAI-1 status that only those patients with low *NOTCH1* mRNA expression may have a higher benefit from adjuvant chemotherapy whilst patients with high *NOTCH1* mRNA expression (22% of the entire cohort, 36% of the uPA/PAI-1 high) have an unfavourable course of disease, suggesting resistance to chemotherapy. This observation can be clinically relevant since adjuvant chemotherapy could be spared for inefficiency in those patients.

*NOTCH1* mRNA has been supposed to be involved in resistance to chemotherapy before by various mechanisms, including epithelial-mesenchymal transition [32], self-renewal of breast cancer stem cells [33] and activation of proliferation pathways [34]. It has been shown that higher *NOTCH1* mRNA expression is associated with decreased paclitaxel effect in breast cancer cell lines [35] or doxorubicin resistance in small-cell lung cancer cells [36]. In addition, it has been shown that endocrine therapy and anti-HER2 therapy may activate the Notch signalling pathway as demonstrated by cell line experiments with tamoxifen by Rizzo et al. [37] and with trastuzumab by Osipo et al. [38].

In preclinical studies with colorectal cancer cell lines, it has been shown that chemosensitivity for 5-FU can be restored by using miRNA for downregulation of the Notch signalling pathway [26]. Another option of Notch downregulation may be established by blocking gamma-secretase as one of the Notch-regulating enzymes. Some drugs for blocking are available (e.g. gamma-secretase-inhibitor, GSI), which may improve the sensitivity to cytotoxic treatment, either chemotherapy [39] or anti-HER2 therapy [40] and endocrine treatment [37]. Particularly, in advanced breast cancer, the GSIs RO-4929097 (in combination with exemestane, *n* = 15 patients) [41] and MK-0752 (in combination with docetaxel, *n* = 30 patients) [42] have been tested in clinical trials (phase I and/or II). The results showed the feasibility of GSIs in combination with cytotoxic treatment, but the drug must be improved due to a wide side effect profile and the small number of patients.

### Strengths and limitations

The PiA-study is a prospective unselected cohort study of consecutively enrolled patients representing the daily clinical routine. Therefore, the overall results with regard to the prognostic impact of *NOTCH1* expression should be considered reliable. However, this is a retrospective subgroup analysis that may be a source of artefacts; therefore, we propose prospective analyses to confirm the predictive value of *NOTCH1* mRNA expression with regard to the chemotherapy effect.

## Conclusions

In this study, with guideline-based adjuvant treatment of patients, *NOTCH1* mRNA expression had a significant prognostic and predictive impact. For patients with high *NOTCH1* mRNA expression, we found an unfavourable course of disease, and in addition, adjuvant chemotherapy seemed to be less effective or even detrimental in these patients. We postulate that particularly patients with high uPA/PAI-1 and simultaneously low *NOTCH1* mRNA expression may benefit from adjuvant chemotherapy.

Considering the substantial impact of *NOTCH1* mRNA expression regarding sensitivity to adjuvant chemotherapy, we propose to study the possibility of downregulating *NOTCH1* by targeted therapies like *γ*-secretase inhibitors.

## Supplementary Information

Below is the link to the electronic supplementary material.

Supplementary file1 (PDF 1806 KB)

## Data Availability

The data generated in this study are available within the article and its supplementary data files. Raw data were generated and processed from the authors and are available on request to the corresponding authors.
