# Correction: Prognostic and predictive impact of NOTCH1 in early breast cancer

**DOI:** 10.1007/s10549-024-07520-6

**Published:** 2024-11-20

**Authors:** Julia Engel, Vanessa Wieder, Marcus Bauer, Sandy Kaufhold, Kathrin Stückrath, Jochen Wilke, Volker Hanf, Christoph Uleer, Tilmann Lantzsch, Susanne Peschel, Jutta John, Marleen Pöhler, Edith Weigert, Karl-Friedrich Bürrig, Jörg Buchmann, Pablo Santos, Eva Johanna Kantelhardt, Christoph Thomssen, Martina Vetter

**Affiliations:** 1 Department of Gynaecology, Martin Luther University Halle-Wittenberg, Halle (Saale), Germany; 2 Institute of Pathology, Martin Luther University Halle-Wittenberg, Halle (Saale), Germany; 3 Onkologische Gemeinschaftspraxis, Fürth, Germany; 4 Department of Obstetrics and Gynaecology, Klinikum Fürth, Nathanstift Fürth, Germany; 5 Gynäkologisch-Onkologische Praxis, Hildesheim, Germany; 6 Present Address: Frauenärzte Am Bahnhofsplatz, Hildesheim, Germany; 7 Department of Gynaecology, Hospital St. Elisabeth and St. Barbara, Halle (Saale), Germany; 8 Department of Gynaecology, St. Bernward Hospital, Hildesheim, Germany; 9 Department of Gynaecology, Helios Hospital Hildesheim, Hildesheim, Germany; 10 Department of Gynaecology, Asklepios Hospital Goslar, Goslar, Germany; 11 Present Address: Department of Gynaecology and Obstretrics, Hospital Wolfenbüttel, Wolfenbüttel, Germany; 12 Institute of Pathology, Hospital Fürth, Fürth, Germany; 13 Present Address: Gemeinschaftspraxis Amberg, Amberg, Germany; 14 Institute of Pathology, Hildesheim, Germany; 15 Institute of Pathology, Hospital Martha-Maria, Halle (Saale), Germany; 16 Institute of Epidemiology, Biometry and Informatics, Martin Luther University Halle-Wittenberg, Halle (Saale), Germany

**Correction: Breast Cancer Research and Treatment** 10.1007/s10549-024-07444-1

The first paragraph of the Results section was incorrectly published with a typo as “NOTCH1 mRNA expression was found to be normally distributed across the tumour tissues of the present Further associations of NOTCH1 mRNA expression were observed with the prognostic markers uPA and PAI-1. Tumours with low uPA and low PAI-1 concentrations (low uPA/PAI-1 status) showed a reduced NOTCH1 mRNA expression. cohort (Supplementary Figure S2). We found no evidence of a relationship between NOTCH1 mRNA expression level and the cellularity of the tumour (ratio tumour cells/stromal cells, Supplementary Figure S3).”

The correct paragraph should appear as “*NOTCH1* mRNA expression was found to be normally distributed across the tumour tissues of the present cohort (Supplementary Figure S2). We found no evidence of a relationship between *NOTCH1* mRNA expression level and the cellularity of the tumour (ratio tumour cells/stromal cells, Supplementary Figure S3).”

The Original article has been corrected.

