# Supplementary material for: Prognostic and predictive impact of NOTCH1 in early breast cancer
Source: Breast Cancer Res Treat. 2024 Aug 17;209(1):27–38. doi: 10.1007/s10549-024-07444-1 (PMC11785638; doi:10.1007/s10549-024-07444-1)
Supplement: Supplementary file 1 — Supplementary file1 (PDF 1806 KB) [file 10549_2024_7444_MOESM1_ESM.pdf]

Prognostic and predictive impact of NOTCH1 in early breast cancer

Julia Engel[1 ORCID: 0000-0002-1518-9518], Vanessa Wieder[1], Marcus Bauer[2 ORCID: 0000-0002-0657-6831], Sandy Kaufhold[1], Kathrin Stückrath[1], Jochen Wilke[3], Volker Hanf[4], Christoph Uleer[5, 6], Tilmann Lantzsch[7], Susanne Peschel[8], Jutta John[9], Marleen Pöhler[10, 11], Edith Weigert[12, 13], Karl-Friedrich Bürrig[14], Jörg Buchmann[15], Pablo Santos[16 ORCID: 0000-0002-3008-014X], Eva Johanna Kantelhardt[1, 16 ORCID: 0000-0001-7935-719X], Christoph Thomssen[#1 ORCID: 0000-0002-9324-2396], Martina Vetter[#, *1 ORCID: 0000-0002-6642-9585]

[1] Department of Gynaecology, Martin Luther University Halle-Wittenberg, Halle (Saale), Germany

[2] Institute of Pathology, Martin Luther University Halle-Wittenberg, Halle (Saale), Germany

[3] Onkologische Gemeinschaftspraxis, Fürth-Roth, Germany

[4] Department of Gynaecology, Nathanstift, Hospital Fürth, Fürth, Germany

[5] Gynäkologisch-Onkologische Praxis, Hildesheim, Germany

[6] Present Address: Frauenärzte am Bahnhofsplatz, Hildesheim, Germany

[7] Department of Gynaecology, Hospital St. Elisabeth and St. Barbara, Halle (Saale), Germany

[8] Department of Gynaecology, St. Bernward Hospital, Hildesheim, Germany

[9] Department of Gynaecology, Helios Hospital Hildesheim, Hildesheim, Germany

[10] Department of Gynaecology, Asklepios Hospital Goslar, 38642 Goslar, Germany

[11] Present Address: Department of Gynaecology and Obstretrics, Hospital Wolfenbüttel, Wolfenbüttel, Germany

[12] Institute of Pathology, Hospital Fürth, 90766 Fürth, Germany

[13] Present Address: Gemeinschaftspraxis Amberg, Amberg, Germany

[14] Institute of Pathology Hildesheim, 31135 Hildesheim, Germany

[15] Institute of Pathology, Hospital Martha-Maria, Halle (Saale), Germany

[16] Institute of Epidemiology, Biometry and Informatics, Martin-Luther-University Halle-Wittenberg, Halle (Saale), Germany

Contributed senior authors

* Correspondence: martina.vetter@uk-halle.de

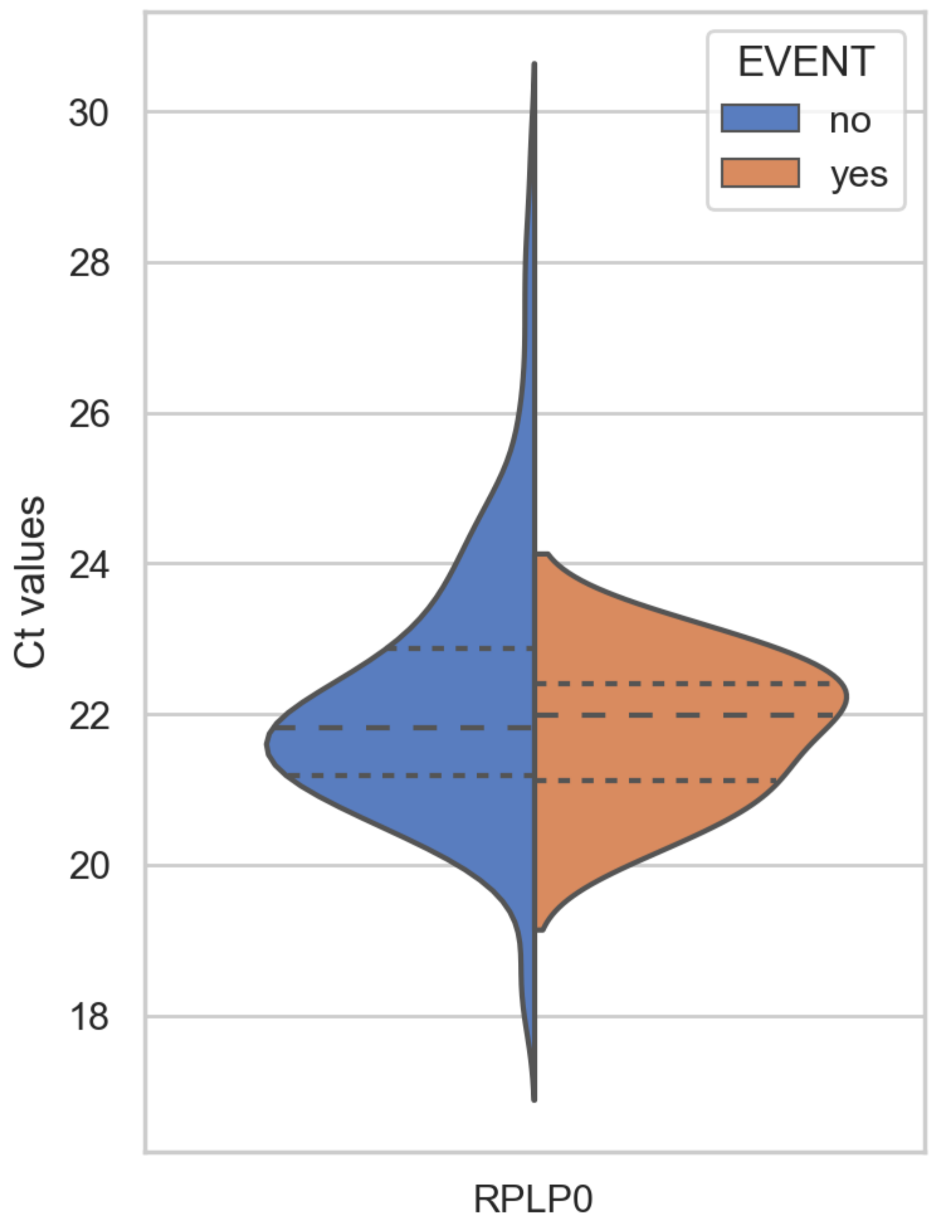

Supplementary Figure S1: Expression of *RPLP0* mRNA among samples of patients with a disease-related event (yes, orange, n=15) and without a disease-related event (no, blue, n=179).
Gene expression does not predict Event (Kolmogorov-Smirnov Test Statistic=0.2123, p-value=0.4954).

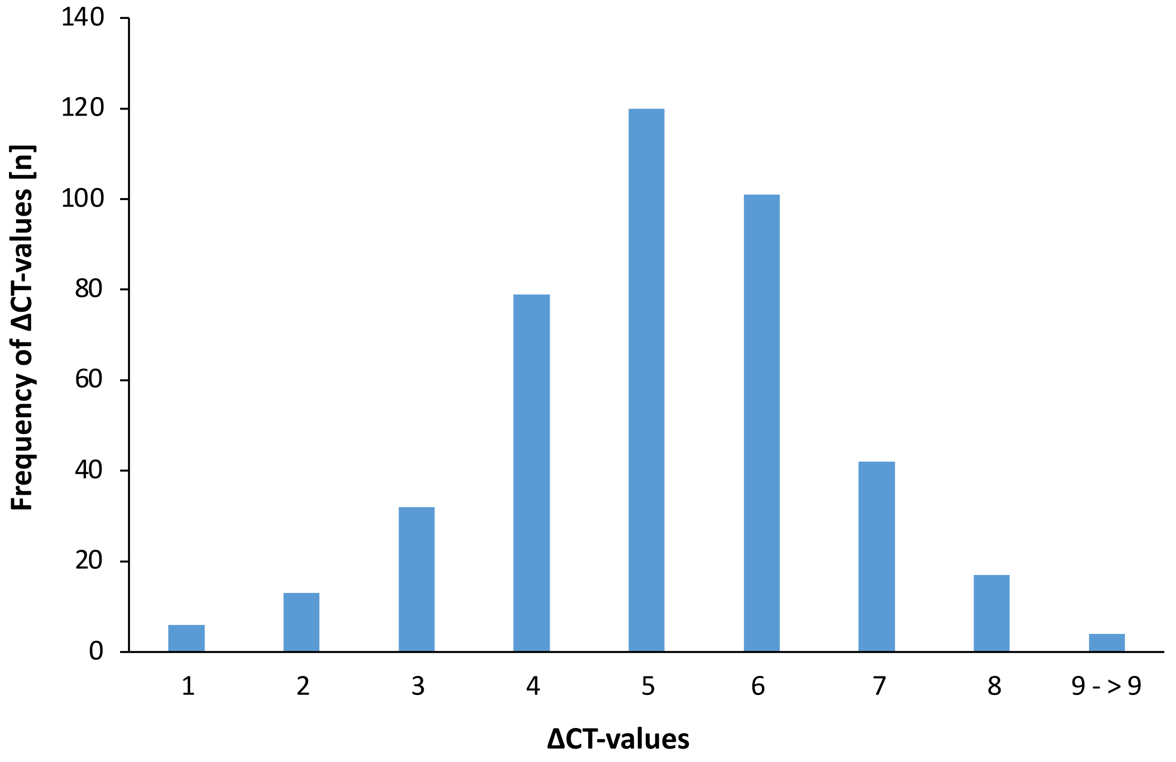

Supplementary Figure S2: Distribution of ΔCt-values (*NOTCH1* Ct-values normalised to the reference gene) within the total cohort

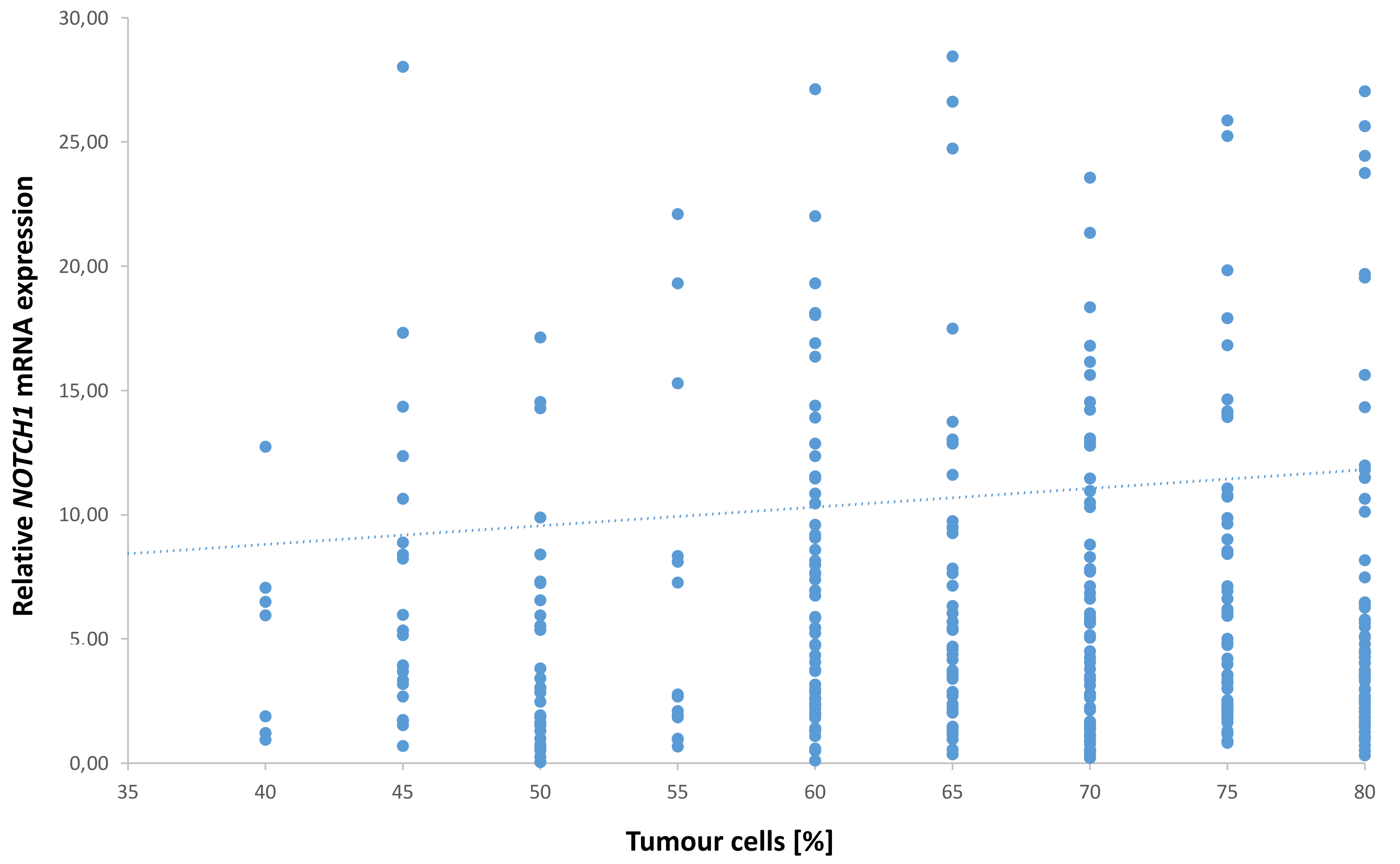

Supplementary Figure S3: Distribution of the relative *NOTCH1* mRNA expression and the amount of tumour cells [%]

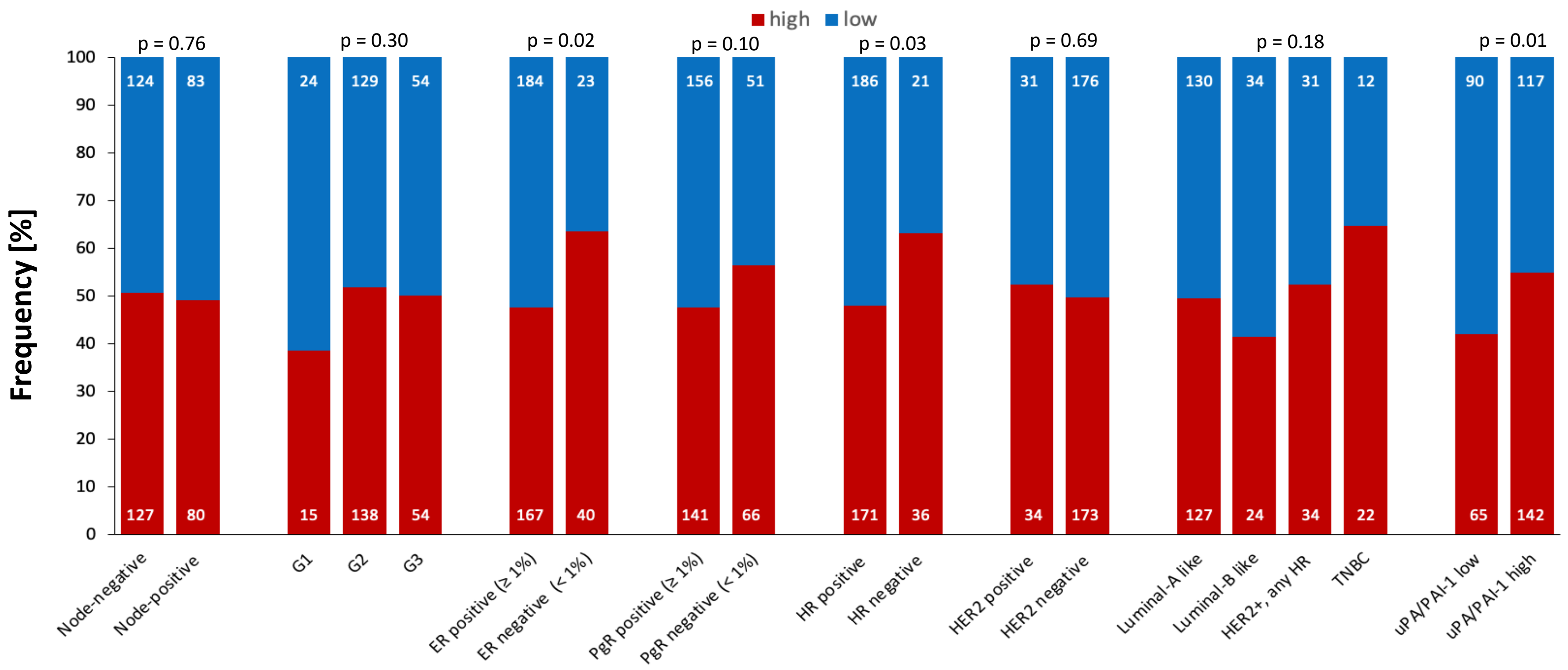

Supplementary Figure S4: Distribution (%) of the relative *NOTCH1* mRNA expression with regard to selected characteristics. The number of patients are given in the bars.

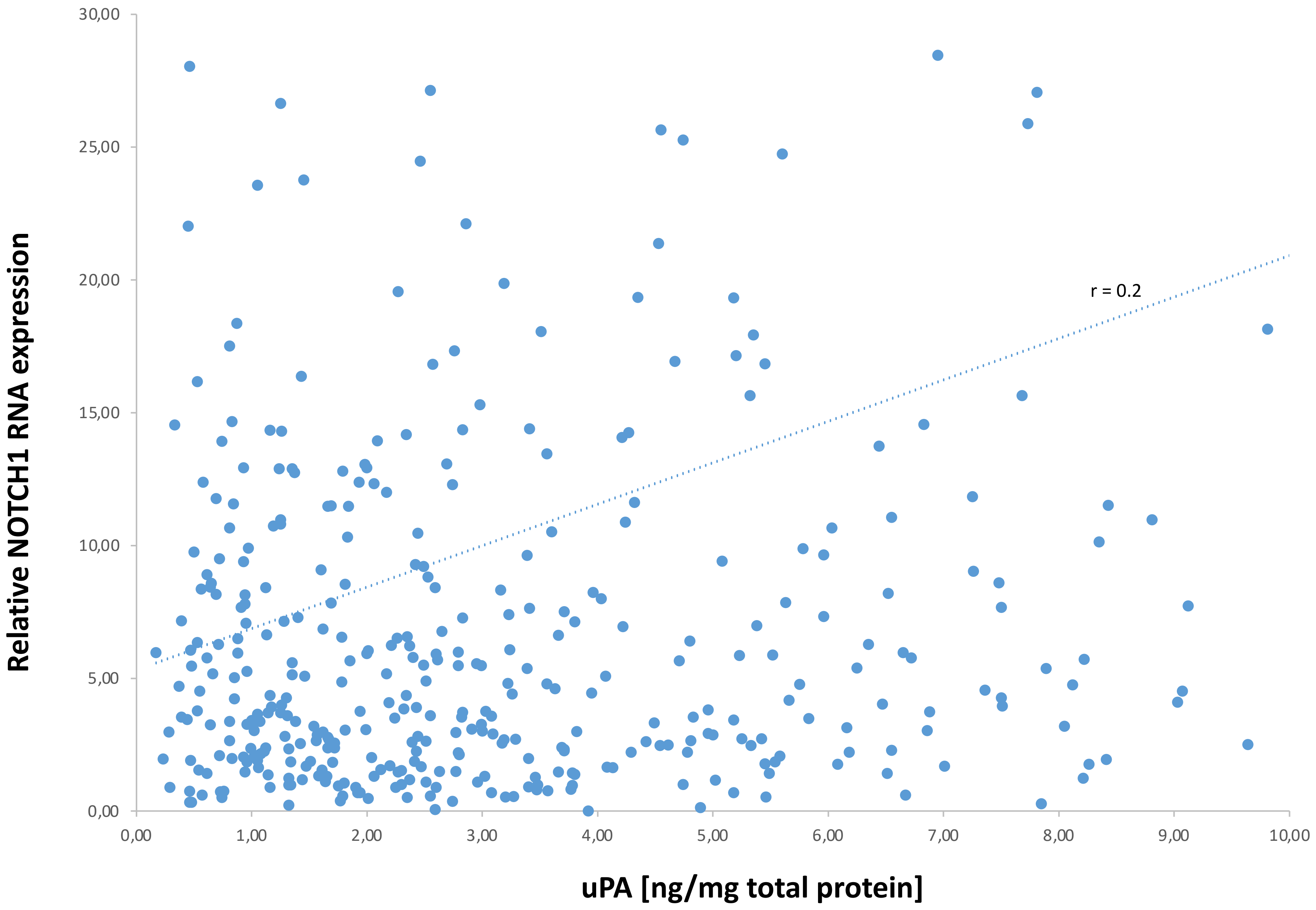

Supplementary Figure S5: Correlation of the *NOTCH1* mRNA expression and uPA protein concentration [ng/mg total protein]

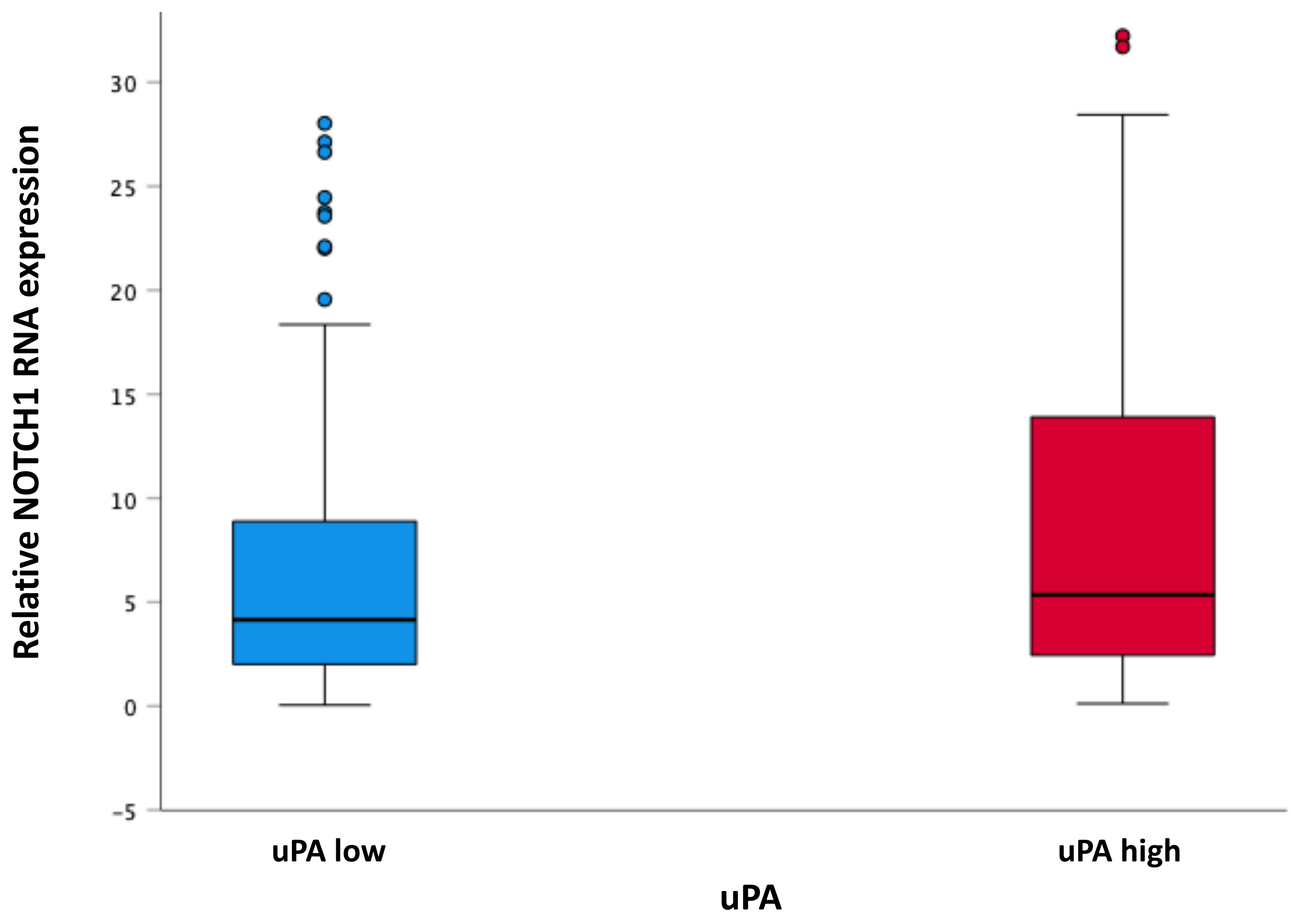

Supplementary Figure S6: Boxplot diagram showing the distribution of relative *NOTCH1* mRNA expression in uPA high and low. Abbreviation: urokinase-type plasminogen activator (uPA), cut off ≥3 ng/mg total protein

A

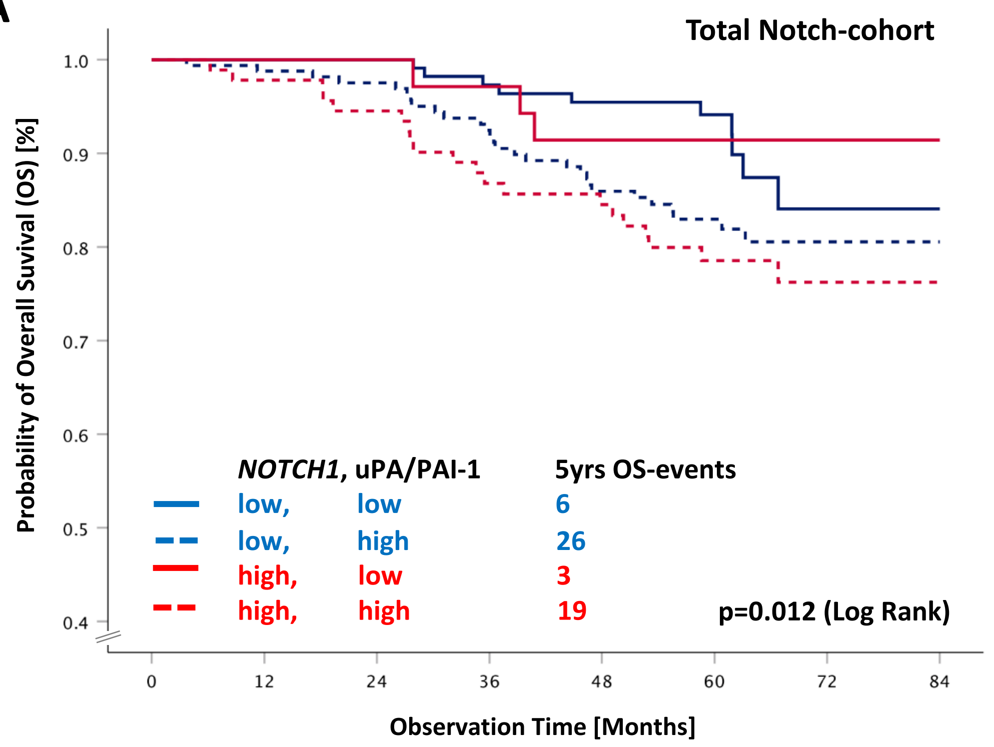

No. at risk	414	403	392	360	338	213	69	33
low, low	120	117	114	107	102	54	17	7
low, high	167	162	157	142	131	83	29	16
high, low	35	35	35	34	31	24	4	2
high, high	92	89	86	77	74	52	19	8

B

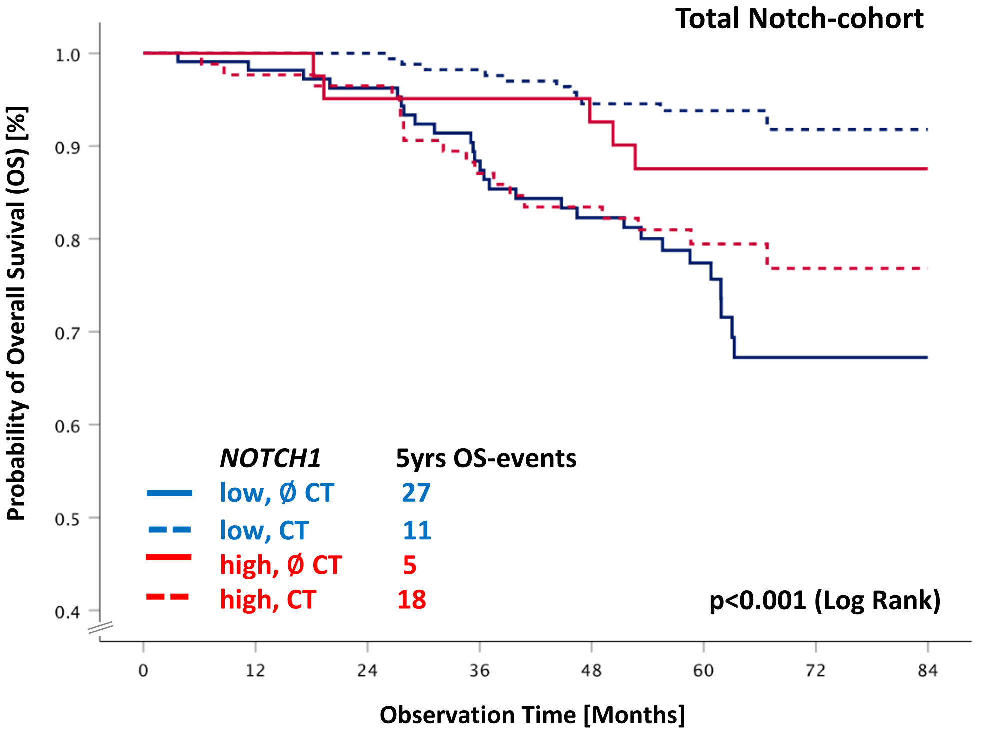

No. at risk	414	403	392	360	338	213	69	33
low, Ø CT	111	107	100	87	79	46	16	7
low, CT	176	172	171	162	154	91	30	16
high, Ø CT	41	41	39	38	37	27	6	1
high, CT	86	83	82	73	68	49	17	9

C

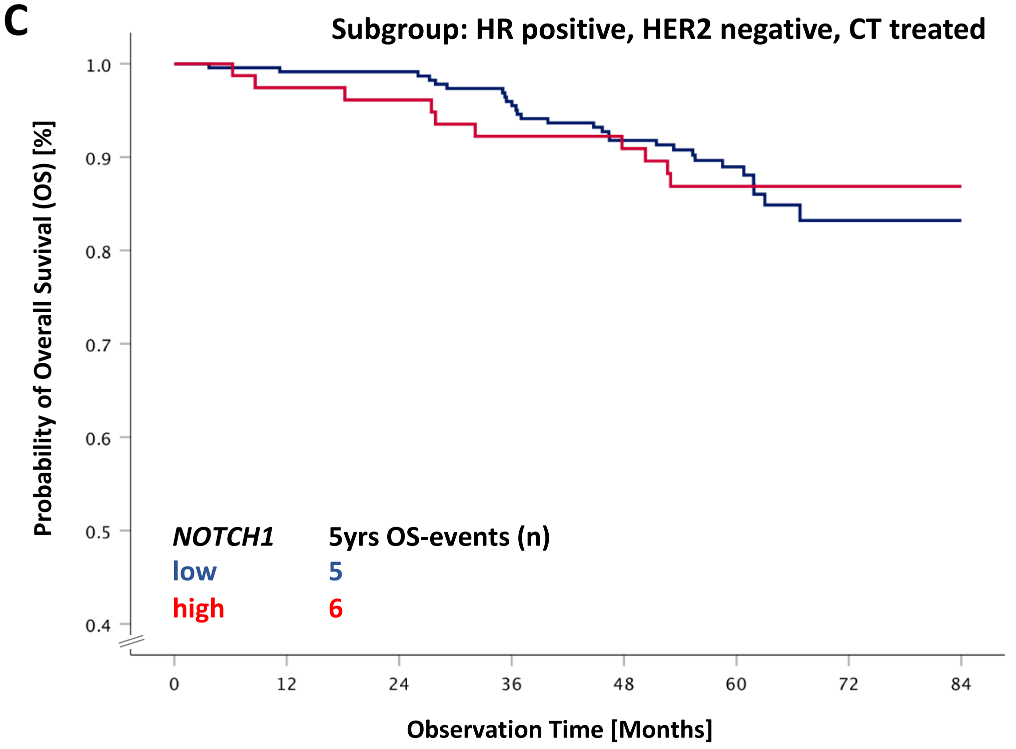

No. at risk	177	171	170	161	155	88	21	6
low	135	132	131	125	120	63	16	6
high	42	39	39	36	35	25	5	0

D

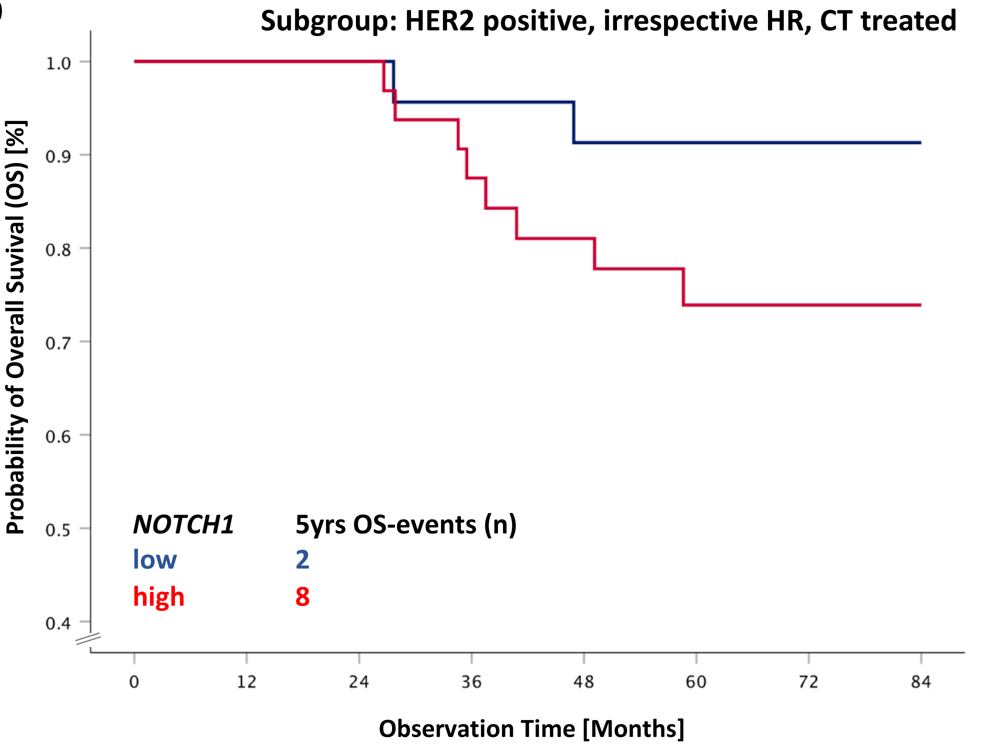

No. at risk	56	55	55	50	46	35	14	6
low	24	23	23	22	21	18	8	4
high	32	32	32	28	25	17	6	2

Supplementary Figure S7: Survival estimates for patients of the Notch-cohort for total Notch-cohort considering *NOTCH1* and uPA/PAI-1 OS (A); total Notch-cohort OS (B); HR positive, HER2 negative, CT treated OS (C); HER2 positive, irrespective HR, CT treated OS (D); the tables present the effective sample size for each interval (no. at risk)

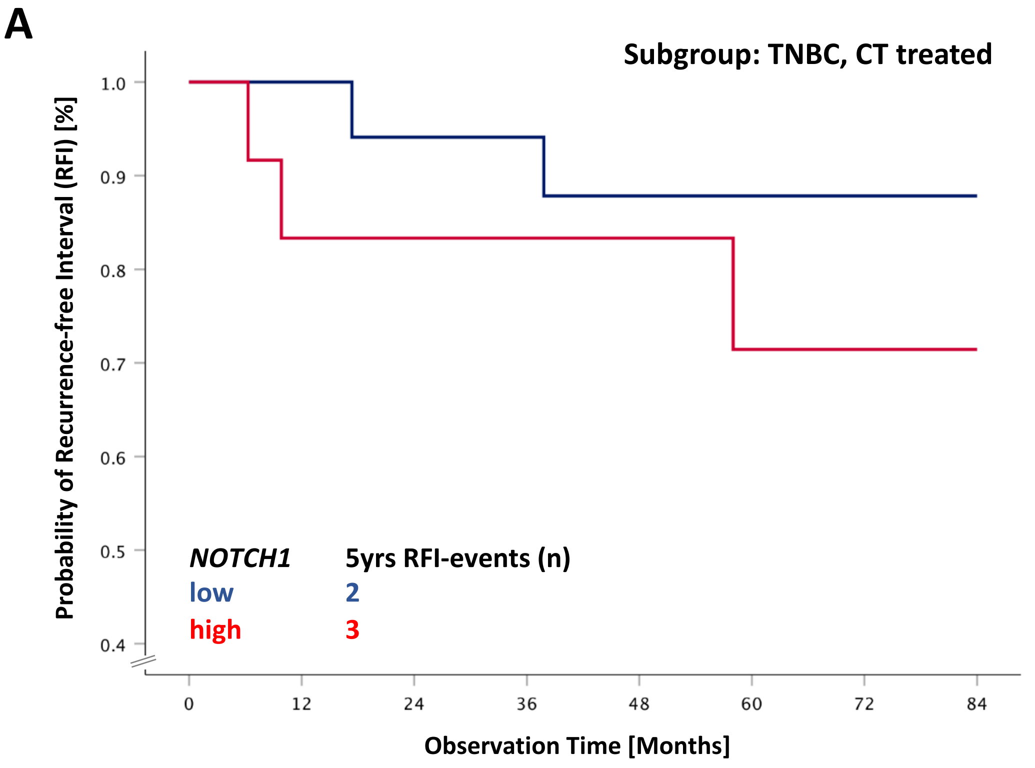

No. at risk	29	27	26	24	21	16	12	9
low	17	17	16	15	13	10	6	4
high	12	10	10	9	8	6	6	5

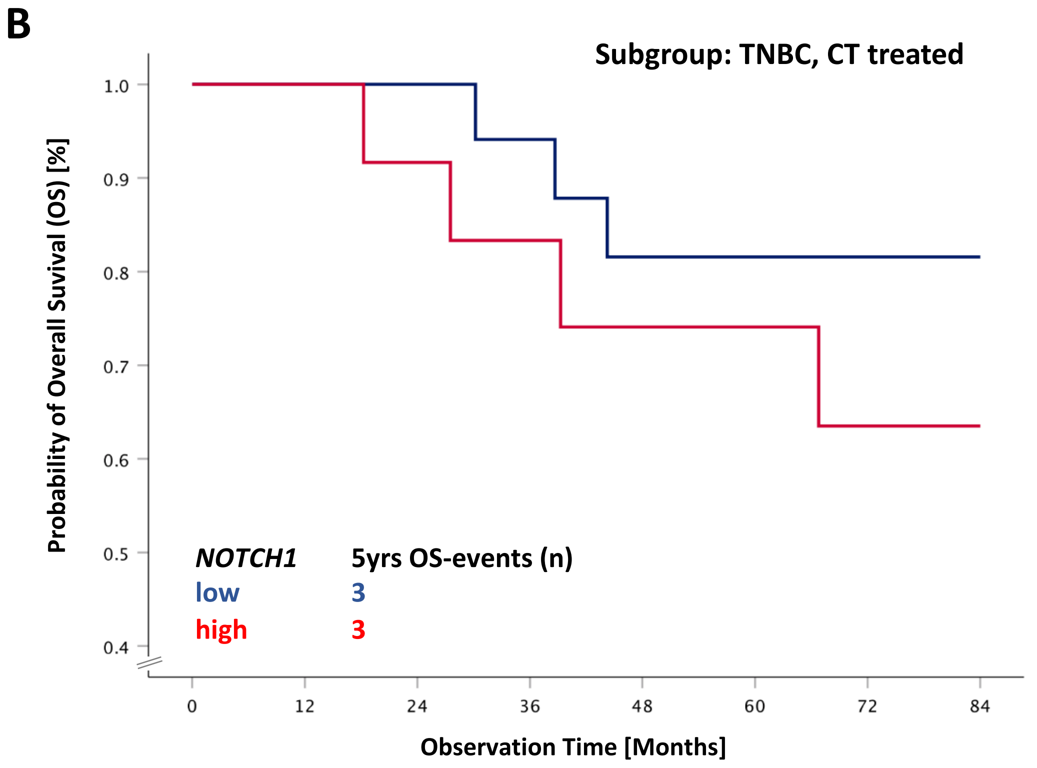

No. at risk	29	29	28	24	21	17	12	9
low	17	17	17	15	13	10	6	4
high	12	12	11	9	8	7	6	5

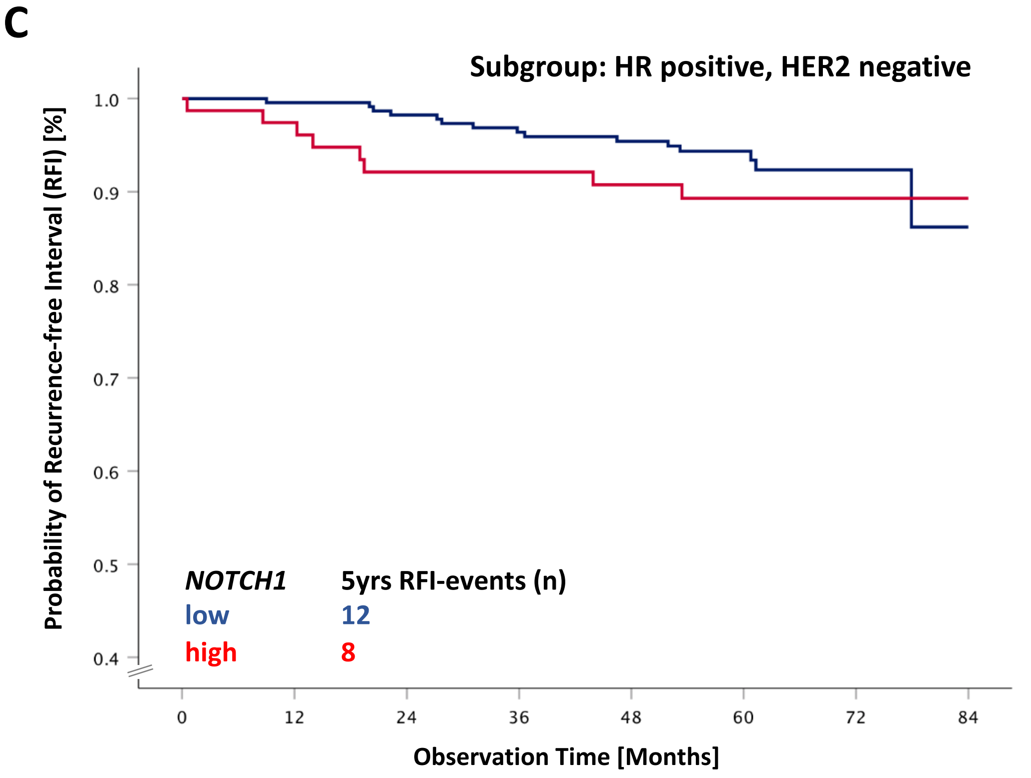

No. at risk	315	304	290	271	257	152	37	12
low	237	230	221	204	192	105	29	11
high	78	74	69	67	65	47	8	1

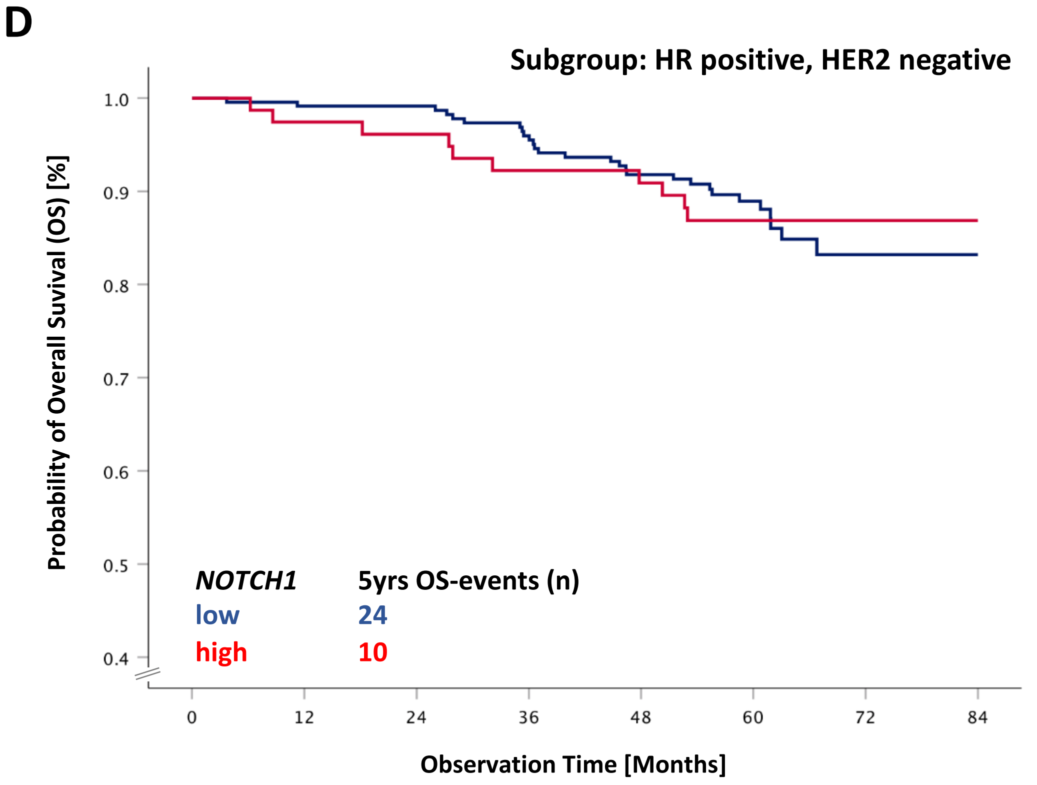

No. at risk	315	305	298	279	263	156	41	15
low	102	230	224	209	195	107	31	13
high	36	75	74	70	68	49	10	2

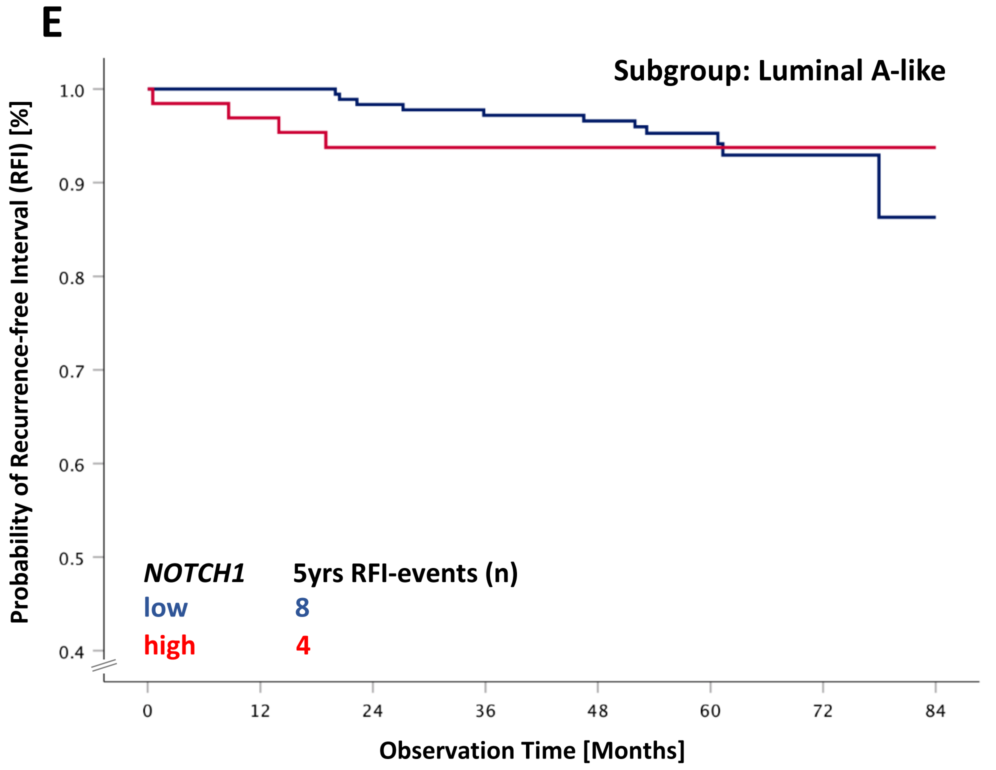

No. at risk	257	250	238	224	214	132	35	11
low	192	188	179	167	158	90	27	10
high	65	62	59	57	56	42	8	1

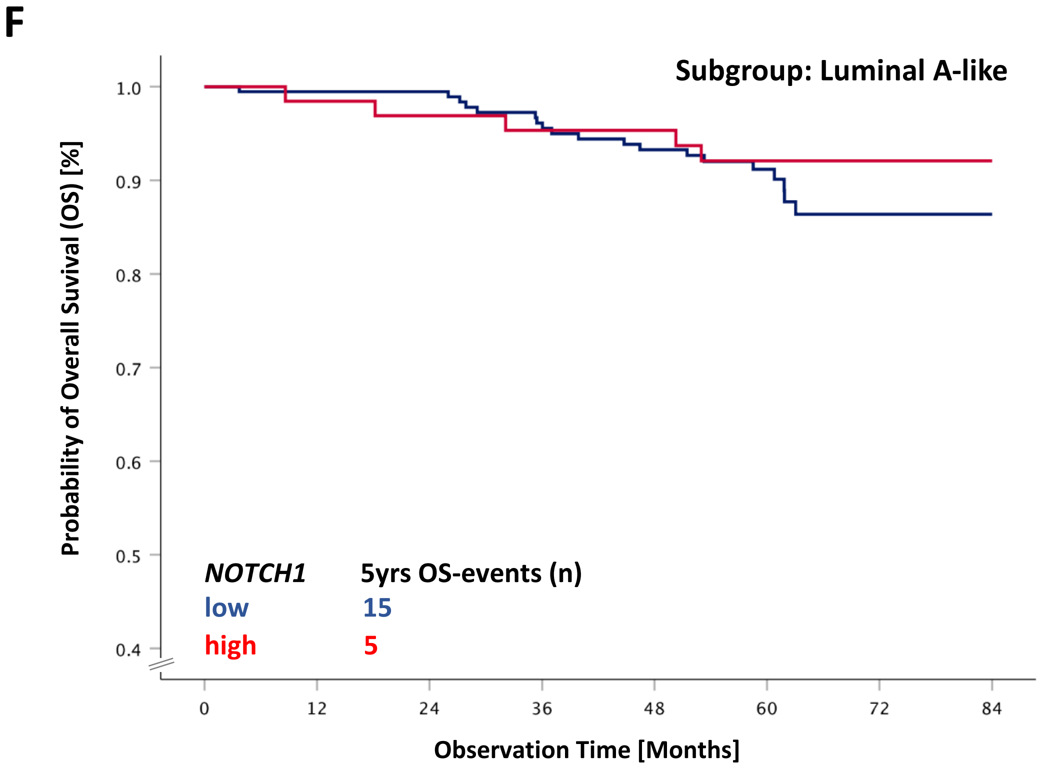

No. at risk	257	251	244	229	219	135	39	14
low	192	188	182	169	160	91	29	12
high	65	63	62	60	59	44	10	2

Supplementary Figure S8: Survival estimates for patients of the Notch-cohort for TNBC, CT treated RFI (A) and OS (B); HR positive, HER2 negative RFI (C) and OS (D); Luminal A-like RFI (E) and OS (F); the tables present the effective sample size for each interval (no. at risk)

A

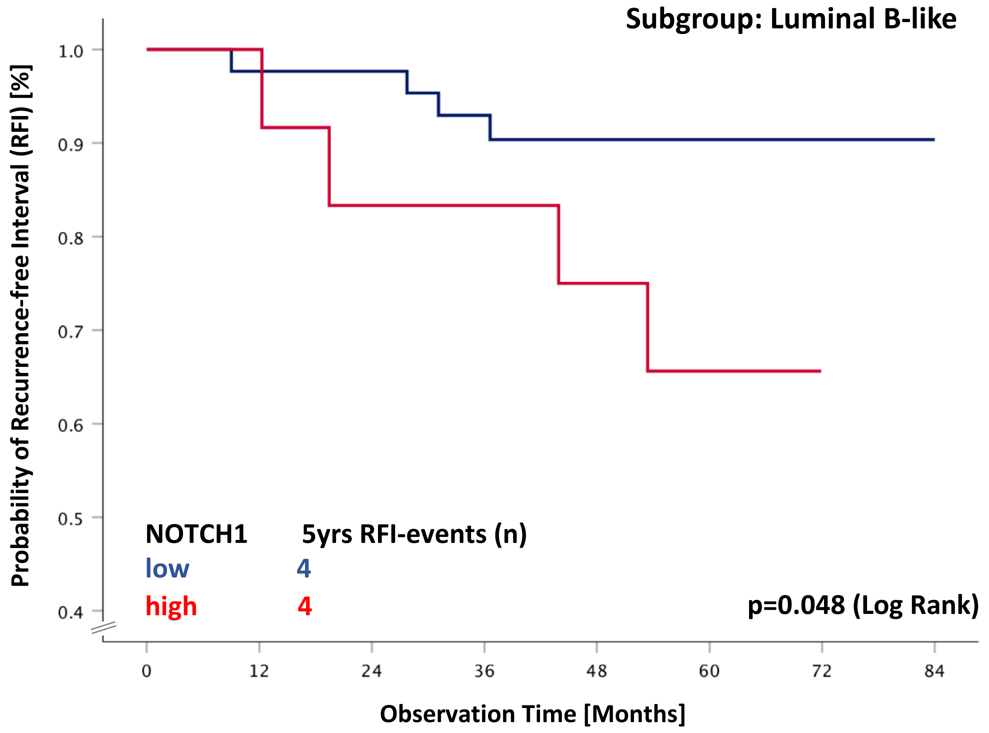

No. at risk	58	54	52	47	43	20	2	0
low	45	42	42	37	34	15	2	0
high	13	12	10	10	9	5	0	0

B

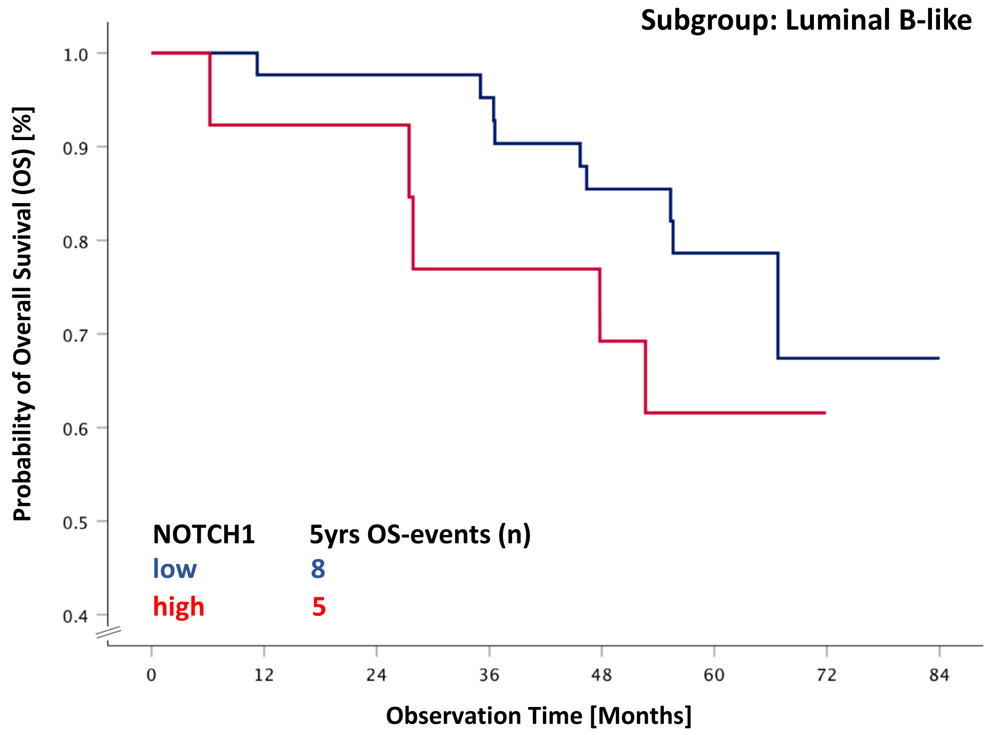

No. at risk	58	54	54	49	44	20	2	0
low	45	42	42	39	35	15	2	0
high	13	12	12	10	9	5	0	0

C

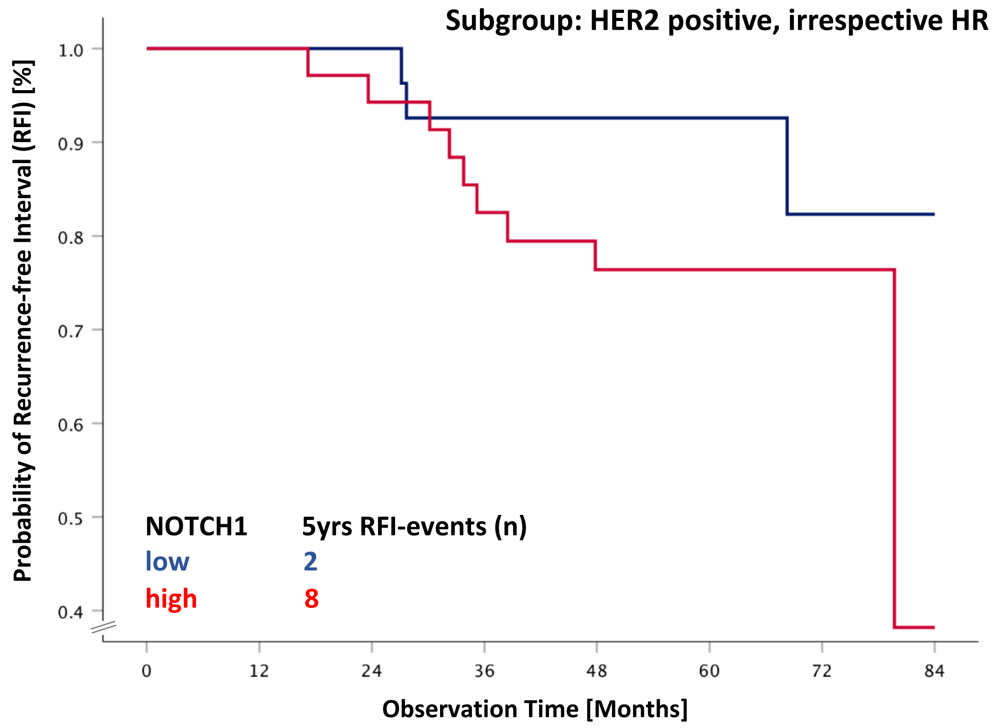

No. at risk	65	63	60	52	48	38	12	2
low	30	29	27	24	23	19	6	2
high	35	34	33	28	25	19	6	0

D

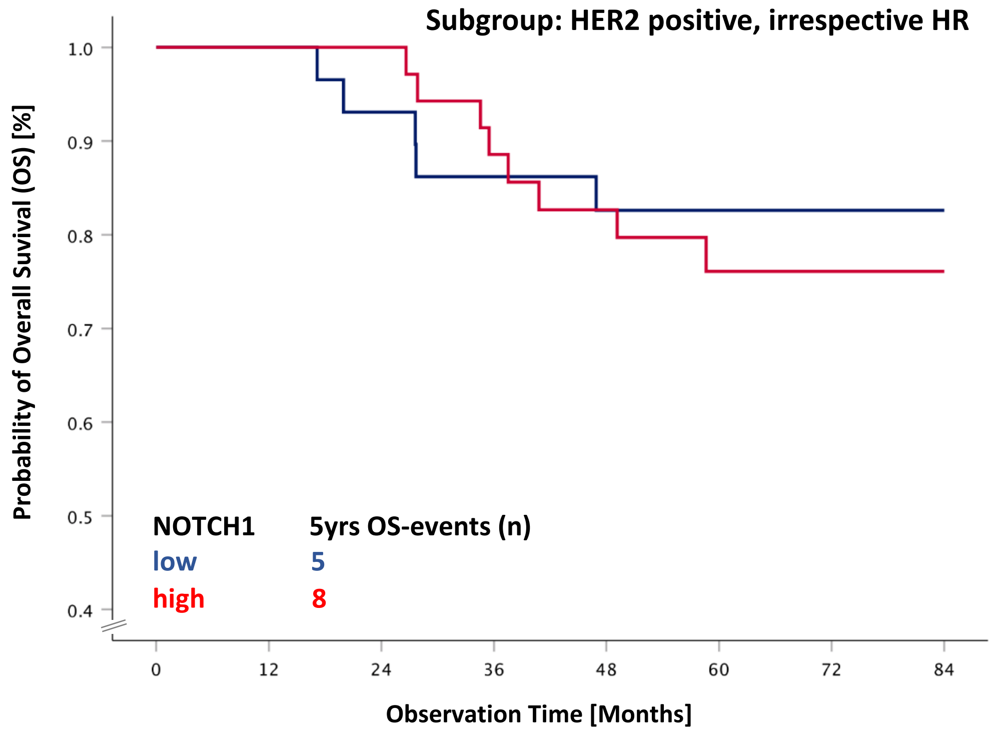

No. at risk	65	64	62	55	51	40	16	7
low	30	29	27	24	23	20	9	5
high	35	35	35	31	28	20	7	2

E

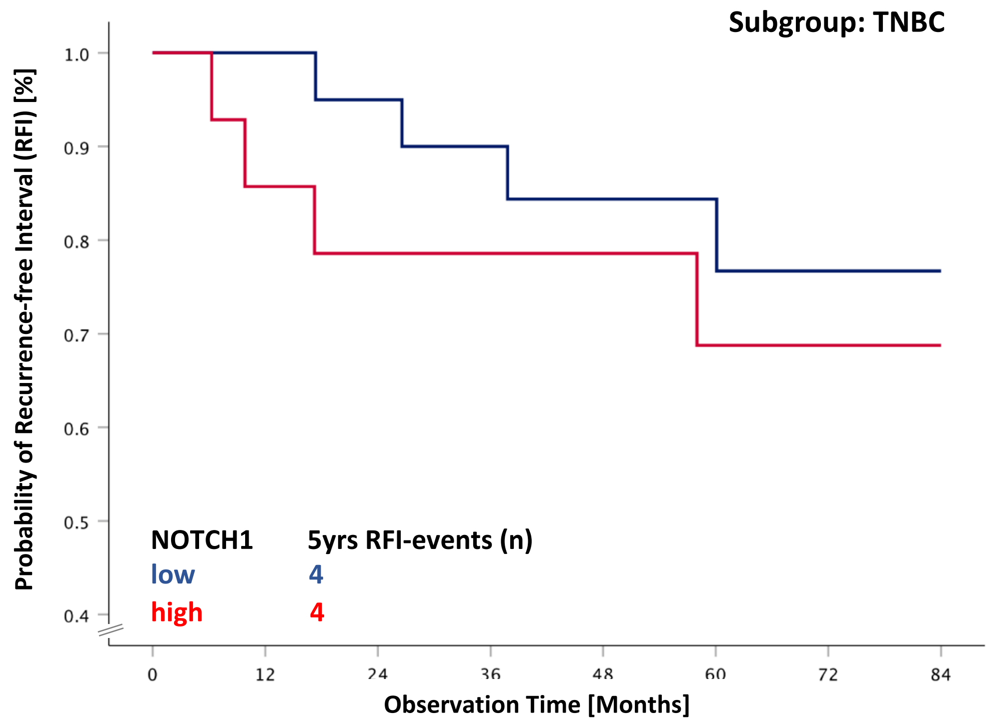

No. at risk	34	32	30	26	23	17	12	9
low	20	20	19	16	14	10	6	4
high	14	12	11	10	9	7	6	5

F

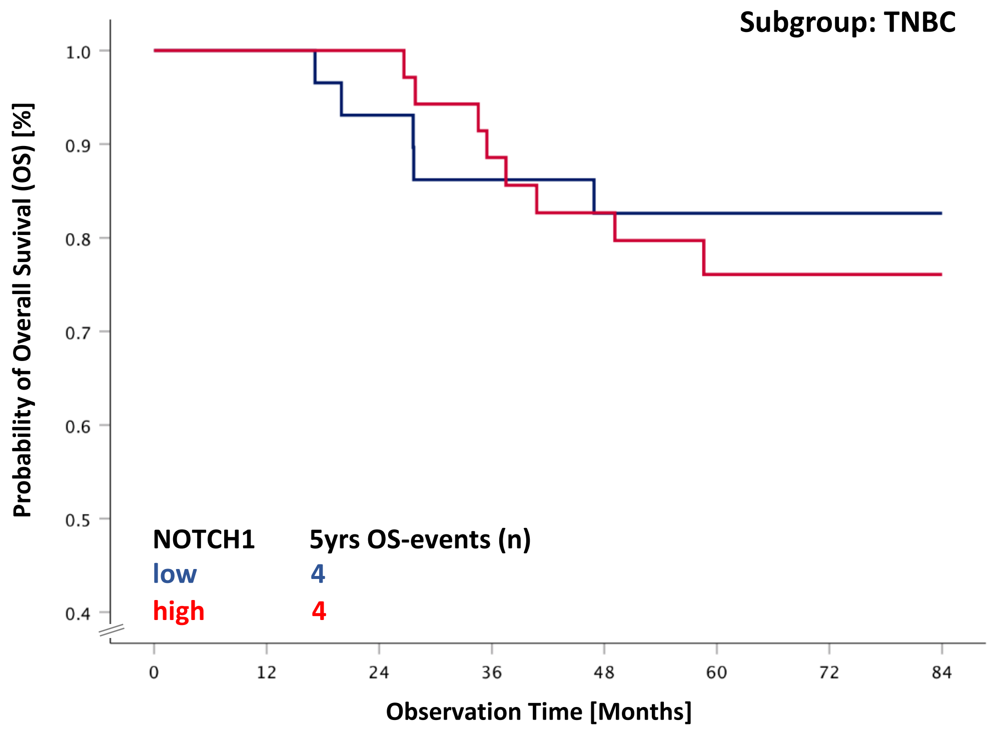

No. at risk	34	34	32	27	24	19	12	9
low	20	20	20	17	15	11	6	4
high	14	14	12	10	9	8	6	5

Supplementary Figure S9: Survival estimates for patients of the Notch-cohort for Luminal B-like for RFI (A) and OS (B); HER2 positive, irrespective HR for RFI (C) and OS (D); TNBC for RFI (E) and OS (F); the tables present the effective sample size for each interval (no. at risk)

Supplementary Table S1: Patients' clinical and histopathological characteristics of the tumours in the total cohort and Notch-cohort

Characteristics	Total cohort	Notch-cohort	p-value χ2
**All**	**1,270**	**414**	
**NOTCH1**			
low		287 (69.32%)	
high		127 (30.68%)	
**Age in yrs**			
< 50	344 (27.09%)	104 (25.12%)	0.273
≥ 50	926 (72.91%)	310 (74.88%)	
**Nodal status**			
Node-negative	780 (61.42%)	251 (60.63%)	0.688
Node-positive	490 (38.58%)	163 (39.37%)	
**Tumour histology**			
ductal (NST)	1039 (81.81%)	329 (79.47%)	0.158
lobular	172 (13.54%)	67 (16.18%)	
others	59 (4.65%)	18 (4.35%)	
**Tumour size**			
≤ 2cm	636 (50.08%)	198 (47.83%)	0.264
> 2cm	634 (49.92%)	216 (52.17%)	
**Grading**			
G1	167 (13.15%)	39 (9.42%)	**0.022**
G2	794 (62.52%)	267 (64.49%)	
G3	309 (24.33%)	108 (26.09%)	
**ER status**			
positive (≥ 1%)	1020 (80.31%)	351 (84.78%)	**0.005**
negative (< 1%)	250 (19.69%)	63 (15.22%)	
**PgR status**			
positive (≥ 1%)	862 (67.87%)	297 (71.74%)	**0.040**
negative (< 1%)	408 (32.13%)	117 (28.26%)	
**HR status**			
positive (ER and/or PgR ≥ 1%)	1038 (81.73%)	357 (86.23%)	**0.004**
negative (ER and PgR < 1%)	232 (18.27%)	57 (13.77%)	
**HER2 status**			
positive	210 (16.54%)	65 (15.70%)	0.578
negative	1060 (83.46%)	349 (84.30%)	
**Biological tumour types**			
Luminal A-like: HR+ HER2-, G1 or G2	777 (61.18%)	257 (62.08%)	**0.001**
Luminal B-like: HR+ HER2-, G3	131 (10.31%)	58 (14.01%)	
HER2+, any HR	200 (16.61%)	65 (15.70%)	
TNBC	152 (11.97%)	34 (8.21%)	
**uPA/PAI-1**			
low: uPA and PAI-1 low	306 (24.09%)	155 (37.44%)	0.949
high: uPA and/or PAI-1 high	509 (40.08%)	259 (62.56%)	
missing	455 (35.83%)	-	

Supplementary Table S2: Distribution of low and high *NOTCH1* mRNA expression in selected characteristics

Characteristics	Notch-cohort	NOTCH1 low	NOTCH1 high	p-value χ2
**All**	**414**	**287**	**127**	
**Age in yrs**				
< 50	104	77 (26.83%)	27 (21.26%)	0.151
≥ 50	310	210 (73.17%)	100 (78.74%)	
**Nodal status**				
Node-negative	251	175 (60.98%)	76 (59.84%)	0.805
Node-positive	163	112 (39.02%)	51 (40.16%)	
**Tumour histology**				
ductal (NST)	329	220 (76.66%)	109 (85.83%)	0.114
lobular	67	52 (18.12%)	15 (11.81%)	
others	18	15 (5.23%)	3 (2.36%)	
**Tumour size**				
≤ 2cm	198	130 (45.30%)	68 (53.54%)	0.140
> 2cm	216	157 (54.70%)	59 (46.46%)	
**Grading**				
G1	39	27 (9.41%)	12 (9.45%)	0.559
G2	267	189 (65.85%)	78 (61.42%)	
G3	108	71 (24.74%)	37 (29.13%)	
**ER status**				
positive (≥ 1%)	351	254 (88.50%)	97 (76.38%)	**0.001**
negative (< 1%)	63	33 (11.50%)	30 (23.62%)	
**PgR status**				
positive (≥ 1%)	297	214 (74.56%)	83 (65.35%)	**0.038**
negative (< 1%)	117	73 (25.44%)	44 (34.65%)	
**HR status**				
positive (ER and/or PgR ≥ 1%)	357	256 (89.20%)	101 (79.53%)	**0.006**
negative (ER and PgR < 1%)	57	31 (10.80%)	26 (20.47%)	
**HER2 status**				
positive	65	30 (10.45%)	35 (27.56%)	**<0.001**
negative	349	257 (89.55%)	92 (72.44%)	
**Biological tumour types**				
Luminal A-like: HR+ HER2-, G1 or G2	257	192 (66.90%)	65 (51.18%)	**<0.001**
Luminal B-like: HR+ HER2-, G3	58	45 (15.68%)	13 (10.24%)	
HER2+, any HR	65	30 (10.45%)	35 (27.56%)	
TNBC	34	20 (6.97%)	14 (11.02%)	
**uPA/PAI-1**				
low: uPA and PAI-1 low	155	120 (41.81%)	35 (27.56%)	**0.006**
high: uPA and/or PAI-1 high	259	167 (58.19%)	92 (72.44%)	

Abbreviations: estrogen receptor (ER), progesteron receptor (PgR), hormone receptor (HR), human epidermal growth factor receptor 2 (HER2), triple-negative breast cancer (TNBC), urokinase-type plasminogen activator (uPA), cut off ≥3 ng/mg total protein, plasminogen activator inhibitor type 1 (PAI-1) cut off ≥14 ng/mg total protein, bold: p-value (Pearson χ2 test) < 0.05

Supplementary Table S3: Univariate odds ratios of low and high *NOTCH1* mRNA expression in selected characteristics

Characteristics	NOTCH1 low (n=287)			NOTCH1 high (n=217)		
	Odds ratio	95% CI	p-value	Odds ratio	95% CI	p-value
**Nodal status**						
negative	1			1		
positive	0.9	0.62-1.46	0.828	1.0	0.68-1.61	0.828
**Tumour size**						
≤ 2cm	1			1		
> 2cm	1.4	0.92-2.12	0.122	0.7	0.47-1.09	0.122
**Grading**						
G1	1			1		
G2	1.1	0.52-2.23	0.842	0.9	0.45-1.93	0.842
G3	0.9	0.39-1.88	0.692	1.2	0.53-2.58	0.692
**ER status**						
positive (≥ 1%)	1			1		
negative (< 1%)	**0.4**	0.24-0.73	**0.002**	**2.4**	1.38-4.11	**0.002**
**PgR status**						
positive (≥ 1%)	1			1		
negative (< 1%)	0.6	0.41-1.01	0.056	1.6	0.99-2.44	0.056
**HR status**						
positive	1			1		
negative	**0.5**	0.27-0.83	**0.009**	**2.1**	1.20-3.76	**0.009**
**HER2 status**						
positive	**0.3**	0.18-0.53	**<0.001**	**3.3**	1.89-5.61	**<0.001**
negative	1			1		
**Biological tumour types**						
Luminal-A like: HR+ HER2-, G1 or G2	1			1		
Luminal-B like: HR+ HER2-, G3	1.2	0.60-2.31	0.647	0.9	0.43-1.68	0.647
HER2+, any HR	**0.3**	0.17-0.51	**<0.001**	**3.4**	1.96-6.05	**<0.001**
TNBC	**0.5**	0.23-1.01	**0.054**	**2.1**	0.99-4.33	**0.054**
**uPA/PAI-1 status**						
low	1			1		
high	**0.5**	0.34-0.83	**0.006**	**1.9**	1.199-2.975	**0.006**

Abbreviations: estrogen receptor (ER), progesteron receptor (PgR), hormone receptor (HR), human epidermal growth factor receptor 2 (HER2), triple-negative breast cancer (TNBC), urokinase-type plasminogen activator (uPA), cut off ≥3 ng/mg total protein, plasminogen activator inhibitor type 1 (PAI-1) cut off ≥14 ng/mg total protein, bold: p-value (Pearson χ^2 test) < 0.05

Supplementary Table S4: Univariate and multivariate analyses of RFI (A) and OS (B) with regard to *NOTCH1* mRNA expression in different groups

A

Groups	sample size (n)	events (n)	RFI event-free [%]	95% CI	univariate hazard ratio	univariate 95% CI	univariate p-value	multivariate hazard ratio	multivariate 95% CI	multivariate p-value
**ALL[a]**										
Low	287	18	93.5	90.56-96.44	1			1		
High	127	20	83.4	76.74-90.06	**2.5**	1.31-4.69	**0.005**	**2.1**	1.08-4.12	**0.029**
**Chemotherapy[b]**										
Low	176	9	94.4	90.87-97.93	1			1		
High	86	17	79.1	70.28-87.92	**4.2**	1.86-9.38	**<0.001**	**3.4**	1.44-7.99	**0.005**
**No chemotherapy[b]**										
Low	111	9	89.9	83.43-96.37	1			1		
High	41	3	92.6	84.56-≥99.9	0.8	0.21-2.93	0.727	0.9	0.23-3.23	0.834
**ALL with regard to chemotherapy[b]**										
Low, no CT	111	9	89.9	83.43-96.37	1.7	0.69-4.41	0.236	**2.9**	1.10-7.39	**0.031**
Low, CT	176	9	94.4	90.87-97.93	1			1		
High, no CT	41	3	92.6	84.56-≥99.90	1.4	0.38-5.15	0.619	2.3	0.62-8.83	0.212
High, CT	86	17	79.1	70.28-87.92	**4.1**	1.81-9.13	**<0.001**	**3.1**	1.32-7.25	**0.009**
**ALL with regard to NOTCH1 and uPA/PAI-1[c]**										
NOTCH1 Low, uPA/PAI-1 low	120	5	95.3	91.18-99.416	1			1		
NOTCH1 Low, uPA/PAI-1 high	167	13	91.0	86.30-95.70	1.9	0.69-5.39	0.214	1.8	0.70-4.63	0.227
NOTCH1 High, uPA/PAI-1 low	35	1	97.1	91.42-≥99.90	0.6	0.07-5.32	0.664	1.1	0.23-5.58	0.888
NOTCH1 High, uPA/PAI-1 high	92	19	78.2	69.38-87.02	**5.2**	1.93-13.92	**0.001**	**3.7**	1.45-9.33	**0.006**
**HR positive, HER2 negative[d]**										
Low	237	12	94.3	91.16-97.44	1			1		
High	78	8	89.3	82.24-96.36	2.03	0.83-4.98	0.120	2.3	0.92-5.54	0.076
**HR positive, HER2 negative and chemotherapy[e]**										
Low	135	6	95.1	91.18-99.02	1			1		
High	42	6	85.0	73.83-96.17	**3.449**	1.11-10.70	**0.032**	**3.6**	1.16-11.17	**0.027**
**HR positive, HER2 negative and no chemotherapy[e]**										
Low	102	6	93.5	88.40-98.60	1			1		
High	36	2	94.3	96.66-≥99.90	0.9	0.18-4.30	0.862	0.8	0.17-4.21	0.840
**HER2 positive, any HR[f]**										
Low	30	2	92.6	82.80-≥99.90	1			1		
High	35	8	76.4	62.09-90.71	2.3	0.700-15.49	0.132	3.3	0.70-15.63	0.129
**HER2 positive, any HR and chemotherapy[f]**										
Low	24	1	95.7	87.27-≥99.90	1			1		
High	32	8	74.1	58.62-89.58	6.5	0.81-51.61	0.079	6.6	0.82-52.61	0.076
**HR negative, HER2 negative**										
Low	20	4	76.7	56.12-97.28	1			-	-	-
High	14	4	68.8	42.73-94.87	2.2	0.49-9.69	0.311			
**HR negative, HER2 negative and chemotherapy**										
Low	17	2	87.8	71.92-≥99.90	1			-	-	-
High	12	3	71.4	43.18-99.62	2.4	0.40-14.43	0.336			

B

Groups	sample size (n)	events (n)	OS [%]	95% CI	univariate hazard ratio	univariate 95% CI	univariate p-value	multivariate hazard ratio	multivariate 95% CI	multivariate p-value
**ALL[a]**										
Low	287	33	87.6	83.48-91.72	1			1		
High	127	22	82.1	75.24-88.96	1.5	0.85-2.50	0.169	1.3	0.75-2.30	0.349
**Chemotherapy[b]**										
Low	176	10	93.6	89.88-97.32	1			1		
High	86	17	79.4	70.58-88.22	**3.6**	1.67-7.97	**0.001**	**2.9**	1.31-6.76	**0.009**
**No chemotherapy[b]**										
Low	111	22	77.4	68.78-86.02	1			1		
High	41	5	87.5	77.31-97.69	0.5	0.19-1.30	0.154	0.5	0.20-1.37	0.184
**ALL with regard to chemotherapy[b]**										
Low, no CT	111	27	77.4	68.97-85.83	**4.2**	1.98-8.75	**<0.001**	**7.1**	3.26-15.25	**<0.001**
Low, CT	176	11	93.8	90.08-97.52	**1**			**1**		
High, no CT	41	5	87.5	77.31-97.69	2.1	0.71-6.06	0.183	**3.6**	1.21-10.86	**0.022**
High, CT	86	18	79.4	70.58-88.22	**3.6**	1.66-7.94	**0.001**	**2.7**	1.20-6.16	**0.016**
**ALL with regard to NOTCH1 and uPA/PAI-1[c]**										
NOTCH1 Low, uPA/PAI-1 low	120	6	94.1	89.40-98.80	1			1		
NOTCH1 Low, uPA/PAI-1 high	167	26	83.0	76.92-89.08	**2.8**	1.21-6.42	**0.016**	**2.6**	1.12-6.02	**0.026**
NOTCH1 High, uPA/PAI-1 low	35	3	91.4	82.19-≥99.90	1.4	0.35-5.24	0.659	1.6	0.41-6.08	0.514
NOTCH1 High, uPA/PAI-1 high	92	19	78.5	69.88-87.12	**3.6**	1.52-8.62	**0.004**	**3.1**	1.31-7.50	**0.010**
**HR positive, HER2 negative[d]**										
Low	237	23	88.9	84.59-93.21	1			1		
High	78	10	86.9	79.26-94.54	1.2	0.57-2.49	0.643	1.3	0.62-2.74	0.479
**HR positive, HER2 negative and chemotherapy[e]**										
Low	135	5	95.8	92.27-99.33	1			1		
High	42	6	85.4	74.62-96.18	**3.9**	1.21-12.95	**0.023**	**3.9**	1.22-13.11	**0.022**
**HR positive, HER2 negative and no chemotherapy[e]**										
Low	102	18	79.6	71.17-88.03	1			1		
High	36	4	88.6	78.02-99.18	0.5	0.17-1.46	0.205	0.5	0.16-1.37	0.165
**HER2 positive, any HR[f]**										
Low	30	5	82.6	68.68-95.52	1			1		
High	35	8	76.1	61.60-90.60	1.3	0.41-3.92	0.665	1.3	0.42-3.92	0.663
**HER2 positive, any HR and chemotherapy[f]**										
Low	24	2	91.3	79.74-≥99.90	1			1		
High	32	8	73.9	58.22-89.58	3.2	0.67-14.95	0.144	3.2	0.68-14.99	0.144
**HR negative, HER2 negative**										
Low	20	4	79.4	61.37-97.43	1			-	-	-
High	14	4	70.4	46.10-94.70	1.7	0.41-6.61	0.478			
**HR negative, HER2 negative and chemotherapy**										
Low	17	3	81.6	62.78-≥99.90	1			-	-	-
High	12	3	74.1	48.82-99.38	1.6	0.32-7.94	0.564			

[a]adjusted to *NOTCH1*, nodal status and biological tumour types, [b]adjusted to *NOTCH1*, nodal status, HR status and HER2 status, [c]adjusted to *NOTCH1*, uPA/PAI-1, nodal status and grading, [d]adjusted to *NOTCH1*, nodal status and grading, [e]adjusted to *NOTCH1* and nodal status, [f]adjusted to *NOTCH1* and HR status, bold: significant; Abbreviations: hormone receptor (HR), human epidermal growth factor receptor 2 (HER2), triple-negative breast cancer (TNBC), urokinase-type plasminogen activator (uPA) cut off ≥3 ng/mg total protein, plasminogen activator inhibitor type 1 (PAI-1) cut off ≥14 ng/mg total protein; bold: p-value (Pearson χ2 test) < 0.05

Supplementary Table S5: Univariate and multivariate analyses of RFI and OS with regard to NOTCH1 RNA expression

		Recurrence-free Interval, 5 years (38 events)							Overall survival, 5 years (55 events)						
**Characteristics**	**sample size**		**Univariate analysis**			**Multivariate analysis**				**Univariate analysis**			**Multivariate analysis**		
	**n=414**	**events**	**hazard ratio**	**95% CI**	**p-value**	**hazard ratio**	**95% CI**	**p-value**	**events**	**hazard ratio**	**95% CI**	**p-value**	**hazard ratio**	**95% CI**	**p-value**
NOTCH 1															
low	287	18	1			1			33	1			1		
high	127	20	**2.5**	1.31-4.69	**0.005**	**2.1**	1.08-4.12	**0.029**	22	1.5	0.85-2.50	0.169	1.3	0.75-2.30	0.349
**Age in yrs**															
< 50	104	12	1.4	0.71-2.79	0.330				9	0.6	0.29-1.19	0.140			
≥ 50	310	26	1						46	1					
**Nodal status**															
negative	251	13	1			1			22	1			1		
positive	163	25	**3.1**	1.61-6.15	**<0.001**	**3.1**	1.57-6.02	**0.001**	33	**2.5**	1.47-4.34	**<0.001**	**2.5**	1.43-4.22	**0.001**
**Tumour histology**															
ductal (NST)	329	35	1						46	1					
lobular	67	3	0.4	0.13-1.40	0.161				8	0.9	0.42-1.87	0.739			
others	18	0	not possible	-	-				1	0.4	0.05-2.63	0.316			
**Tumour size**															
< 2cm	198	12	1						11	1					
≥ 2cm	216	26	**2.2**	1.09-4.28	**0.028**				44	**3.9**	2.06-7.71	**<0.001**			
**Grading**															
G1, G2	306	19	1						29	1					
G3	108	19	**3.1**	1.62-5.79	**<0.001**				26	**2.8**	1.63-4.70	**<0.001**			
**Biological tumour types**															
Luminal A-like: HR+ HER2- G1 or G2	257	12	1			1			21	1			1		
Luminal B-like HR+ HER2- G3	58	8	**3.1**	1.28-7.69	**0.012**	**3.1**	1.24-7.49	**0.015**	13	**2.9**	1.50-5.98	**0.002**	**2.84**	1.42-5.68	**0.003**
HER2+, any HR	65	10	**3.4**	1.46-7.82	**0.004**	**2.8**	1.17-6.59	**0.021**	13	**2.5**	1.26-5.03	**0.009**	**2.33**	1.14-4.75	**0.020**
TNBC	34	8	**5.4**	2.21-13.23	**<0.001**	**4.7**	1.91-11.75	**<0.001**	8	**3.2**	1.39-7.11	**0.006**	**3.03**	1.33-6.90	**0.008**

Abbreviations: hormone receptor (HR), human epidermal growth factor receptor 2 (HER2), confidence interval (CI); bold: significant in prognostic parameters
